# Gene therapy prevents onset of mitochondrial cardiomyopathy in neonatal mice with Ndufs6 deficiency

**DOI:** 10.1038/s41420-025-02524-7

**Published:** 2025-05-22

**Authors:** Xiaoxian Zhang, Li Huang, Cheng Li, Jinjuan Yang, Fuyu Duan, Qiang Su, Yuelin Zhang, Meng Kou, Xiaoya Zhou, Liyan Guo, Shaoxiang Chen, Yongxia Niu, Ziyue Li, Sihua Ou, Min Zhang, Kenneth King-Yip Cheng, Jianlong Wu, Xiang Xu, Qizhou Lian

**Affiliations:** 1 Prenatal Diagnostic Center and Cord Blood Bank, Guangzhou Women and Children’s Medical Centre, Guangzhou Medical University, Guangzhou, China; 2 Faculty of Synthetic Biology, Shenzhen University of Advanced Technology; CAS Key Laboratory of Quantitative Synthetic Biology, Shenzhen Institutes of Advanced Technology, Chinese Academy of Sciences, Shenzhen, China; 3 Department of Pharmacy, Shenzhen Clinical Research Center for Neurological Diseases, The First Affiliated Hospital of Shenzhen University, Shenzhen Second People’s Hospital, Shenzhen, China; 4 Department of Pain Medicine, Huazhong University of Science and Technology Union Shenzhen Hospital, Shenzhen, China; 5 Department of Emergency Medicine, Guangdong Provincial People’s Hospital, Guangdong Academy of Medical Sciences, Southern Medical University, Guangzhou, China; 6 Department of Health Technology and Informatics, The Hong Kong Polytechnic University, Hong Kong SAR, China; 7 State Key Laboratory of Trauma and Chemical Poisoning, Department of Stem Cell and Regenerative Medicine, Daping Hospital, Army Medical University, Chongqing, China; 8 Center for Translational Stem Cell Biology, Hong Kong, and State Key Laboratory of Pharmaceutical Biotechnology, The University of Hong Kong, Hong Kong SAR, China

**Keywords:** Heart failure, Preclinical research

## Abstract

Mutations in genes affecting mitochondrial complex I (CI) can lead to mitochondrial cardiomyopathy (MCM) yet no effective treatment. This study sought to determine whether adeno-associated virus 9 (AAV9)-based gene therapy could prevent or rescue Ndufs6 deficiency-induced MCM at different disease stages. Using Ndufs6^gt/gt^ mice to mimic MCM, cardiac dysfunction was evident at week 4 post-birth, showing reduced ejection fraction, CI activity, increased fibrosis, mitochondrial fission, and disrupted cristae. Neonatal and adult mice were intravenously given AAV9-hNdufs6 (1e14 vg kg^−1^). AAV9-hNdufs6 therapy effectively prevented neonatal mice’s cardiac dysfunction onset, preserving CI activity and cristae structure for 11 months. In contrast, therapy in adult mice post-disease onset failed to reverse or halt progression of heart dilation and failure after 3 months, showing mitochondrial abnormalities and cardiomyocyte apoptosis. Mechanistically, adult mouse Kupffer cells demonstrated enhanced phagocytic capabilities compared to neonatal mice, with higher expression levels of AAV9 cell surface receptors observed in neonatal mouse hearts, rendering neonatal mice more responsive to AAV9-mediated gene therapy for heart tissue. Additionally, AAV9-hNdufs6 gene therapy initiated at an early stage increased Ndufs6 expression in cardiac tissue, preserved mitochondrial structure and function, prevented cardiomyocyte fibrosis through modulation of the AMPK/Drp1 signaling pathway. In conclusion, early intervention with AAV9-hNdufs6 gene therapy can effectively prevent the onset of MCM, but intervention after disease onset has limited efficacy.

## Introduction

Mitochondrial diseases are caused by the mutations of mitochondrial DNA (mtDNA) or nuclear DNA (nDNA) that affect mitochondrial function. It is estimated that the prevalence of this disease is about 1 in 8500 births [1]. Of these conditions, mitochondrial complex I (CI) deficiency is a prominent cause of cardiomyopathy in children, accounting for about 30% of diagnosed cases [2]. As the major component of the mitochondrial respiratory chain (MRC), CI transfers electrons during oxidative phosphorylation (OXPHOS) and contributes to cellular energy production. CI deficiency can cause various symptoms, especially affecting the central nervous system, heart, liver, and skeletal muscles [3–6]. Accumulating evidence indicates that CI deficiency also poses a high risk of reactive oxygen species (ROS) overproduction in cardiomyopathy [7–9]. Mitochondrial CI consists of more than 44 distinct protein subunits that are tightly assembled within mitochondria. While the precise functions of these subunits remain unclear, genetic defects in some have been linked to cardiomyopathy. Ndufs6, also known as nicotinamide adenine dinucleotide (NADH) dehydrogenase iron-sulfur (Fe-S) protein 6, is a subunit of mitochondrial CI. As part of the Fe-S cluster in CI, Ndufs6 transfers electrons from NADH to ubiquinone, generating a proton gradient across the mitochondrial inner membrane that is essential for adenosine triphosphate (ATP) synthesis. The Ndufs6 ortholog in Y. lipolytica yeast binds zinc at the interface of two functional modules within the enzyme complex, which is critical for CI biogenesis [10]. Mice lacking Ndufs6 in cardiac tissue exhibited severe mitochondrial cardiomyopathy (MCM) caused by oxidative stress [11]. Defects in Ndufs6 can result in mitochondrial dysfunction with an estimated prevalence of 1 in 590,000 births [1, 12]. Despite of a low incidence, the clinical outcome is fatal. In humans, Ndufs6 deficiency was first discovered by sequencing of two cell lines from patients with mitochondrial CI disorders in 2004 [13]. Then, more reports demonstrated there is a wide clinical spectrum of NDUFS6-deficiency related disease, including aging, neuropathy and cardiovascular disorders [14–16]. Currently, there is no cure for CI deficiency-induced cardiomyopathy except for heart transplantation. However, the transplantation is a complex and costly procedure with poor accessibility due to donor shortages. Novel therapeutic approaches are urgently needed to treat CI deficiency-induced cardiac conditions.

Over the past decade, adeno-associated virus (AAV)-mediated gene replacement therapy has been extensively researched and has demonstrated promising therapeutic potential in various gene mutation-induced disorders [17–20]. In a preclinical study, systemic delivery of AAV-mediated frataxin significantly prevented and reversed the cardiac dysfunction and ultrastructure changes in Friedreich ataxia, a type of MCM [21]. Cardiac dysfunction was also prevented and/or reversed following AAV therapy in a mouse model of X-linked Barth syndrome [22]. Furthermore, AAV9-Plakophilin 2 (PKP2) administration restored PKP2 protein function in both induced pluripotent stem cell-derived cardiomyocytes and a mouse model [19]. AAV gene therapy shows great promise in preventing and treating mitochondrial-related cardiomyopathy. However, the therapeutic potential and mechanism of AAV gene therapy for CI-deficiency-induced MCM remain unclear. Therefore, the current study aimed to verify the therapeutic efficacy of AAV-mediated human Ndufs6 (AAV-hNdufs6) gene therapy for CI cardiomyopathy. In particular, the therapeutic timeframe was explored for prophylactic and symptomatic therapy with neonatal and adult mice model, respectively. Ndufs6 cardiac-specific deficient mouse model (Ndufs6^gt/gt^-mice) was used in our study, and AAV9 was chosen as a vector for delivering hNdufs6 to cardiac tissues with superior transduction efficiency. Our findings are expected to confirm the therapeutic efficacy and timeframe of AAV9-hNdufs6 gene therapy in conditions with CI cardiomyopathy. Our exploration on the underlying mechanism also shed a new light on the novel therapeutic targets for further studies.

## Results

### Ndufs6^gt/gt^ mice mimic CI-deficient cardiomyopathy well with Ndufs6 deficiency

**Fig. 1:**
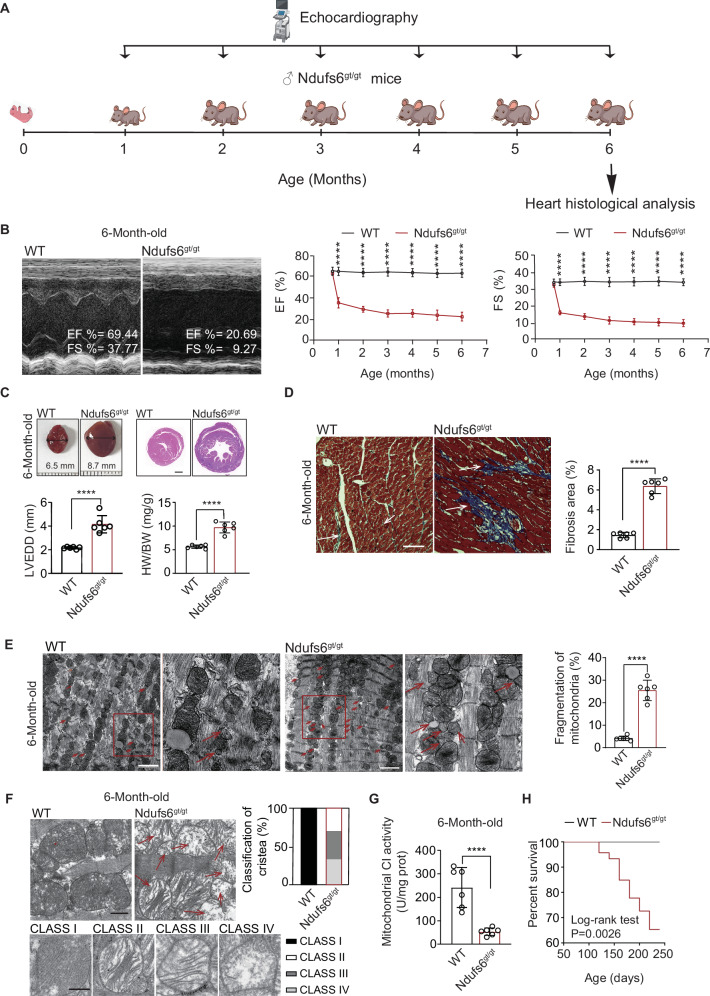
Ndufs6 knock out in heart tissue could develop cardiomyocyte in mice. **A** Procedure for identification of Ndufs6^gt/gt^ mice. **B** Representative images of left ventricular echocardiograph in WT and Ndufs6^gt/gt^ mice and M-mode echocardiograph data (EF% and FS%) of left ventricle in WT and Ndufs6^gt/gt^ mice (*n* = 6). **C** Representative images of heart and cardiac cross section with H&E staining from WT and Ndufs6^gt/gt^ mice, followed by statistical analysis of LVEDD and HW/BW (*n* = 6). Scale bar = 1 mm. **D** Representative images of Masson’s trichrome staining and quantitative measurement of heart fibrosis (*n* = 6). Scale bar = 100 μm. **E** Representative images of mitochondrial ultrastructure and quantification of mitochondrial fragmentation (*n* = 6). Scale bar = 2 μm. **F** Representative images of mitochondrial cristae and the quantification of the degree of cristae damage (*n* = 6). Scale bar = 500 nm. **G** Mitochondrial CI activity of WT and Ndufs6^gt/gt^ group (*n* = 6). **H** Survival rate of WT and Ndufs6^gt/gt^ groups (*n* = 15).

Monthly echocardiography was performed on both wild type (WT) and Ndufs6^gt/gt^ mice during 1 to 6 months postnatal (Fig. 1A). Analysis of echocardiography results revealed cardiac dysfunction in Ndufs6^gt/gt^ mice at one month postnatal based on ejection fraction (EF) and fractional shortening (FS), progressing to severe heart failure since3 months postnatal (Fig. 1B). Compared to WT mice, histological analysis revealed significant cardiac dilation with increasing left ventricular end-diastolic diameter (LVEDD) and heart/body weight ratio (HW/BW) in Ndufs6^gt/gt^ mice at 6 months postnatal (Fig. 1C). The significantly increased fibrotic area in Ndufs6^gt/gt^ mice indicated severe fibrosis in cardiac tissues (Fig. 1D). Additionally, transmission electron microscopy (TEM) revealed abnormal mitochondrial construction with increased fission and cristae damage in cardiomyocytes from Ndufs6^gt/gt^ mice compared to WT mice (Fig. 1E, F). The activity of mitochondrial oxidation respiratory chain CI showed a significant decrease in the Ndufs6^gt/gt^ mice compared with WT mice (Fig. 1G). The lifespan of Ndufs6^gt/gt^ mice was also significantly shorter (Log-rank test *P* = 0.0026, Fig. 1H) with growth retardation in body weight after 10 weeks postnatal (Fig. S1A). Ndufs6 expression deficiency was confirmed in Ndufs6^gt/gt^ mice through genotyping and assessment of mRNA expression in cardiac tissues (Fig. S1B, C). Differentially expressed genes (DEGs) analysis showed 186 genes were down-regulated in the cardiac tissues of Ndufs6^gt/gt^ mice compared with those of WT adult mice (Fig. S1D), which were involved in a variety of metabolic processes according to GO enrichment, including anaerobic respiration, fatty acid metabolism, ROS metabolism process, respiration and oxidative phosphorylation (Fig. S1E). The KEGG enrichment results indicated that the knockout of *Ndufs6* gene in heart may be involved in cardiac-related diseases, such as diabetic cardiomyopathy and cardiac muscle contraction. Furthermore, the downregulation of the *Ndufs6* gene was associated with alterations in the AMPK signaling pathway and fatty acid metabolism (Fig. S1F). Therefore, Ndufs6^gt/gt^ mice could serve as an ideal model to mimic CI-deficient cardiomyopathy.

### Prophylactic administration of AAV9-hNdufs6 prevented cardiac dysfunction and prolong the lifespan in asymptomatic Ndufs6^gt/gt^ mice

**Fig. 2:**
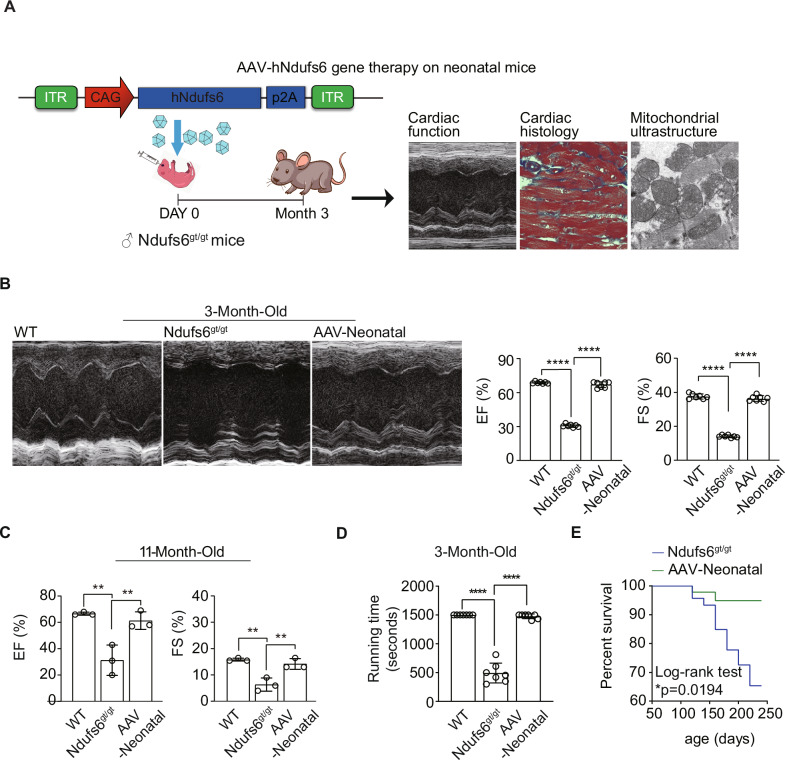
Administration of AAV9-hNdufs6 prevented cardiac dysfunction in newborn Ndufs6^gt/gt^ mice. **A** Schematic diagram of prophylactic gene therapy for neonatal Ndufs6^gt/gt^ mice. **B** Representative M-mode echocardiograms and quantification of left ventricular EF% and FS% for 3-month-old AAV neonatal therapy mice (*n* = 7). **C** The quantification of M-mode echocardiograms of left ventricular EF% (WT vs. Ndufs6^gt/gt^, **p = 0.0033; Ndufs6^gt/gt^ vs. AAV-Neonatal, **p = 0.0074) and FS% (WT vs. Ndufs6^gt/gt^, **p = 0.0021; Ndufs6^gt/gt^ vs. AAV-Neonatal, **p = 0.0055) for 11-month-old WT, Ndufs6^gt/gt^ and AAV-Neonatal mice (*n* = 3). **D** Treadmill exercise tolerance test in 3-month-old AAV neonatal treatment group (*n* = 7). **E** Survival rate of Ndufs6^gt/gt^ and AAV-Neonatal group (n = 15). (*p = 0.0194).

Prior to the study intervention, the delivery efficiency of AAV9 to the target organ was assessed in two-month-old mice. Robust enhanced green fluorescent protein (eGFP) protein expression was observed in the liver, heart, brain, and spleen 30 days after AAV9-CAG-eGFP injection (Fig. S2A–C, *n* = 4). Prophylactic treatment was given to asymptomatic neonatal mice at postnatal day (PND) 0 based on the predicted development of cardiomyopathy in Ndufs6^gt/gt^ mice. Specifically, newborn mice received AAV9-hNdufs6 at a dose of 1e14 vg·kg^-1^ or the same volume of PBS via the orbital venous plexus. Cardiac function was evaluated through echocardiography and a treadmill tolerance test three months post injection, followed by tissue collection for molecular and histological analysis (Fig. 2A). The administration of AAV9-hNdufs6 significantly preserved cardiac function in neonatal Ndufs6^gt/gt^ mice at 3 months post-injection with EF of 66.9 ± 2.6% and FS of 36.4 ± 1.7% (Fig. 2B). The therapeutic benefits could be lasted up to 11 months (Fig. 2C) with stable EF (61.3 ± 6.7%) and FS (28.3 ± 4.0%). Besides, a treadmill test recorded the running performance of each mouse over 1500 s (slope, 15.0°; speed, 25 km·h^-1^). Mice treated with AAV9-hNdufs6 (AAV-Neonatal) were able to run for the full 1500 s, while no Ndufs6^gt/gt^ mouse could persist for more than 800 s, with some unable to continue after just 300 s (Fig. 2D). Therefore, better exercise tolerance was indicated in Ndufs6^gt/gt^ mice receiving prophylactic AAV9-hNdufs6 therapy. Overall, a prolonged lifespan was observed for Ndufs6^gt/gt^ mice with therapy (Log-rank test *P* = 0.0194, Fig. 2E), which attributed to stable cardiac function and better exercise tolerance.

### Prophylactic AAV9-hNdufs6 alleviated fibrosis, apoptosis and mitochondrial damage in Ndufs6-deficient cardiomyopathy

**Fig. 3:**
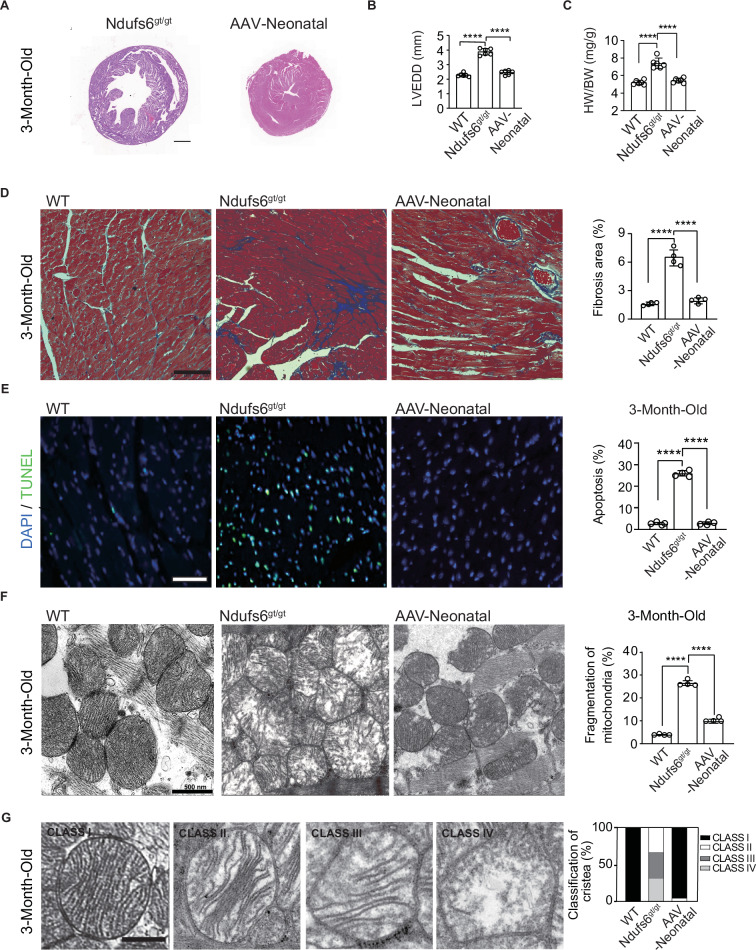
Administration of AAV9-hNdufs6 prevented pathological changes in newborn Ndufs6^gt/gt^ mice. **A** Representative images illustrating the morphology of heart. Scale bar = 1 mm. **B** Quantitative analysis of LVEDD of WT, Ndufs6^gt/gt^ and AAV-Neonatal mice at 3 months old (*n* = 6). **C** Quantitative analysis of HW/BW ratio of WT, Ndufs6^gt/gt^ and AAV-Neonatal mice at 3-months old (*n* = 6). **D** Representative images of Masson’s trichrome staining and quantitative measurement of heart fibrosis (n = 4). Scale bar = 100 μm. **E** Representative images of TUNEL staining and quantification analysis of the heart tissue from WT, Ndufs6^gt/gt^ and AAV-Neonatal mice (*n* = 4). Scale bar = 50 μm. The tissue slides utilized are consecutive sections from the same specimen stained with Masson’s trichrome on (**D**). **F** Representative mitochondrial ultrastructure and quantitative measurement of mitochondrial fragmentation in different experimental groups (*n* = 4). Scale bar = 1 μm. **G** Representative images of mitochondrial cristae morphology and the quantification of the degree of cristae damage, scale bar = 500 nm in WT, Ndufs6^gt/gt^ and AAV-Neonatal groups. The results were calculated based on mitochondrial classification system (*n* = 4, with at least 100 mitochondria averaged for each replicate).

**Fig. 4:**
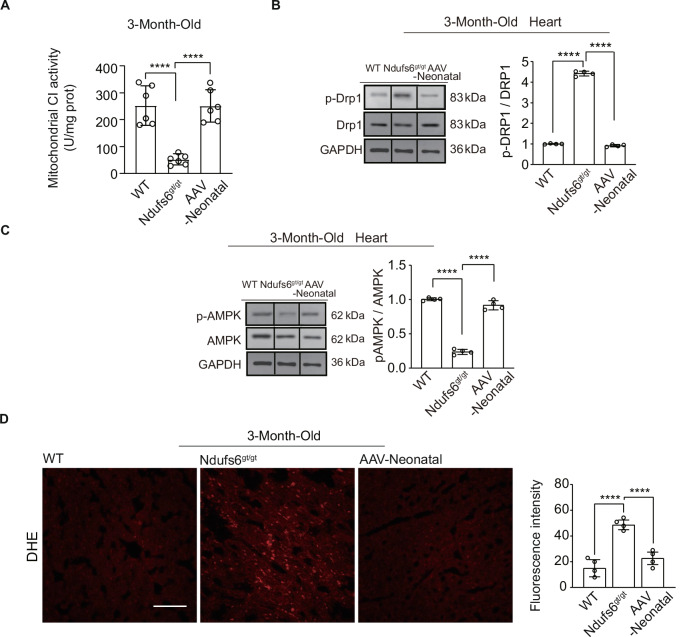
Administration of AAV9-hNdufs6 prevented cardiomyopathy in Ndufs6^gt/gt^ mice via the AMPK pathway. **A** Cardiac mitochondrial CI activity results of WT, Ndufs6^gt/gt^, and AAV-Neonatal groups (n = 6). **B** Protein expression levels of p-Drp1, Drp1, p-AMPK and AMPK in cardiac tissue (expressed as relative fold, normalized to β-Actin and followed by normalization to WT gray scale/ β-Actin gray scale) were analyzed in the WT, Ndufs6^gt/gt^, and AAV-Neonatal groups (**B**, **C**). Quantitative assessment of p-Drp1/Drp1 (**B**) and p-AMPK/AMPK (**C**) expressions in WT mice, Ndufs6^gt/gt^ and AAV-Neonatal mice is presented (*n* = 4). **D** DHE staining for ROS and quantification are illustrated (*n* = 4). Scale bar = 50 μm.

Three months after administration, cardiac tissue and blood serum were collected for histochemical and molecular studies. Cardiomegaly was not observed in AAV-Neonatal mice following prophylactic treatment with AAV9-Ndufs6, consistent with previous findings (Fig. 3A). Ndufs6^gt/gt^ mice exhibited obvious cardiac hypertrophy, while no significant differences were observed in LVEDD (Fig. 3B) or the HW/BW ratio between AAV-Neonatal and WT mice (Fig. 3C). AAV9-hNdufs6 significantly prevented myocardial hypertrophy and maintained normal cardiac tissue morphology in neonatal mice. Additionally, Masson’s trichrome staining revealed that AAV9-hNdufs6 treatment could prevent myocardial fibrosis, with AAV-Neonatal mice showing less fibrosis area (1.9 ± 0.1%) compared with Ndufs6^gt/gt^ groups (6.5 ± 0.7%, Fig. 3D). Cardiac dilation, hypertrophy, and fibrosis were markedly reduced in AAV-neonatal mice. Subsequently, we used the Terminal Deoxynucleotidyl Transferase mediated dUTP Nick-End Labeling (TUNEL) assay to investigate if AAV gene therapy could prevent apoptosis. A significant number of apoptotic cells were present in Ndufs6^gt/gt^ mice compared to WT and AAV-Neonatal mice (Fig. 3E), indicating the potential of AAV9-hNdufs6 treatment to prevent cardiomyocyte apoptosis in asymptomatic Ndufs6^gt/gt^ mice. In order to evaluate the impact of Ndufs6 deficiency on mitochondrial dynamics in cardiac tissue, we examined myocardial mitochondrial ultrastructure using transmission electron microscopy scanning. In AAV9 treated Ndufs6^gt/gt^ mice, mitochondrial fission was prevented, as evidenced by comparable results of mitochondrial fragmentation in AAV-Neonatal and WT mice (Fig. 3F). The severity of mitochondrial ultrastructure damage was assessed and classified into four levels, from class I to IV (Fig. 3G). Ndufs6^gt/gt^ mice showed severe mitochondrial disruption with damaged cristae (Class II, 33.3%; Class III, 36.0%; Class IV, 30.7%), while AAV-Neonatal mice exhibited a normal structure of mitochondrial cristae (Class I, 96.0%; Class II, 4.0%) (Fig. 3G). The measurement of mitochondrial CI activity in cardiac tissues showed a significant decrease in the Ndufs6^gt/gt^ group compared with the WT, which was reversed after AAV9-hNdufs6 treatment in AAV-Neonatal groups (Fig. 4A). Therefore, prophylactic AAV9-hNdufs6 therapy could restore Ndufs6 expression, so as to prevent cardia dysfunction by alleviating apoptosis and mitochondrial damage in cardiomyocytes.

### The AMPK/Drp1 signaling pathway was involved in molecular mechanism of prophylactic AAV9-hNdufs6 therapy

Mitochondrial-related proteins were assessed by Western blot with GAPDH normalization using cardiac tissue collected 3 months post gene therapy. The expression of p-Drp1, a regulator of mitochondrial fission, significantly increased in Ndufs6^gt/gt^ mice but was dramatically reversed after prophylactic AAV9-hNdufs6 therapy in AAV-Neonatal mice (Fig. 4B). Furthermore, we investigated the activation status of AMPK, an upstream regulator of Drp1 in mitochondrial dynamics [23, 24]. Compared with WT mice, a marked reduction in AMPK phosphorylation (p-AMPK) was detected in Ndufs6^gt/gt^ mice, which was prevented after prophylactic AAV9-Ndufs6 therapy in AAV-Neonatal mice (Fig. 4C). Furthermore, mitochondrial ROS product was detected using dihydroethidium (DHE) staining, which was a well-known major cause of cardiomyocyte apoptosis and mitochondrial damage and involved in the crosstalk with AMPK and Drp1. Compared with Ndufs6^gt/gt^ controls, the ROS product in AAV-Neonatal mice decreased to a similar level with WT mice after prophylactic AAV9-Ndufs6 therapy (Fig. 4D). Taken together, these data demonstrate that prophylactic administration of AAV9-hNdufs6 could prevent cardiomyopathy by reducing ROS, and the AMPK/Drp1 pathway was involved.

### AAV9-hNdufs6 therapy had limited effect to relieve the deterioration of cardiomyopathy in adult Ndufs6^gt/gt^ mice

**Fig. 5:**
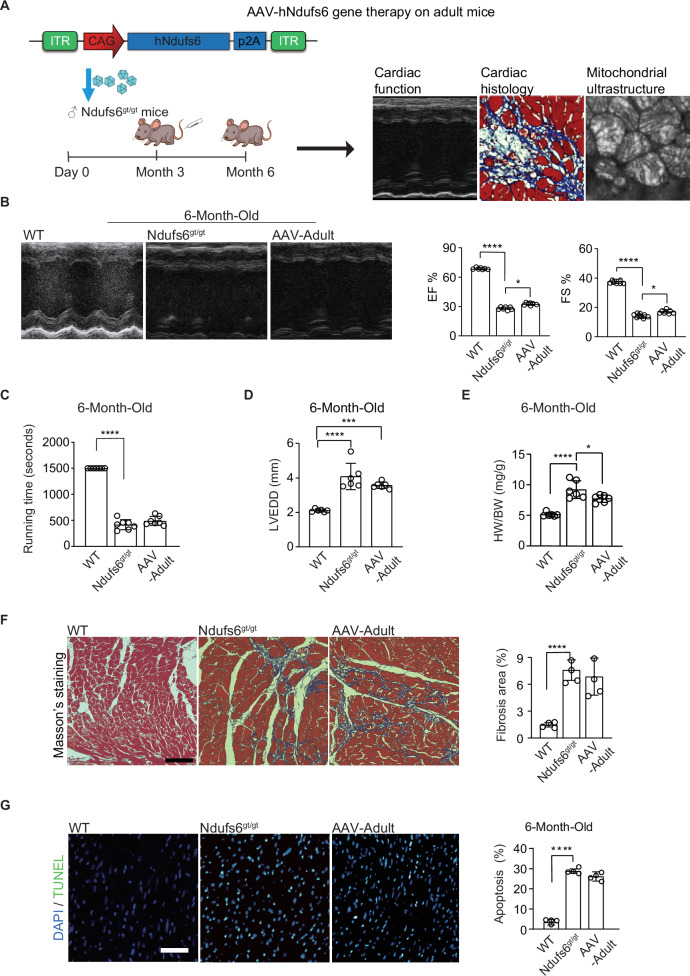
Administration of AAV9-hNdufs6 could not reverse cardiac dysfunction in adult Ndufs6^gt/gt^ cardiomyopathy mice. **A** Schematic diagram of gene therapy for adult Ndufs6^gt/gt^ mice. **B** Representative M-mode echocardiograms and quantification of left ventricular EF% (*p = 0.0134) and FS% (*p = 0.0222) (*n* = 7). **C** Running time of treadmill exercise tolerance test for WT, Ndufs6^gt/gt^, and AAV-Adult groups (*n* = 7). **D** Quantification of LVEDD for WT, Ndufs6^gt/gt^, and AAV-Adult groups (***p = 0.0001) (*n* = 6). **E** Quantification of HW/BW for WT, Ndufs6^gt/gt^, and AAV-Adult groups (*p = 0.0377) (*n* = 6). **F** Representative images of Masson’s trichrome staining and measurement of fibrosis (*n* = 4). Scale bar = 100 μm. **G** Representative images of TUNEL staining for apoptosis and quantification analysis of cardiomyocytes from WT, Ndufs6^gt/gt^, and AAV-Adult mice (*n* = 4). Scale bar = 50 μm. The tissue slides utilized are consecutive sections from the same specimen stained with Masson’s trichrome on (**F**).

In clinic, many patients remain undiagnosed until they develop mitochondrial cardiomyopathies. To simulate this clinical situation, we evaluated the therapeutic efficacy of AAV9-hNdufs6 in adult Ndufs6^gt/gt^ mice (approximately 3 months old) to explore the potential benefits of AAV gene therapy given after disease onset. We assessed cardiac function using echocardiography and treadmill tolerance testing 3 months after injecting AAV9-hNdufs6 via the tail vein at a dose of 1e14 vg·kg^-1^. Molecular and histological analyses were conducted on cardiac tissue and blood serum (Fig. 5A). Echocardiography showed decreased cardiac function in Ndufs6^gt/gt^ mice, while a slight improvement was observed in AAV-Adult group (Fig. 5B). However, no significant improvement on running time was detected in exercise tolerance tests for the AAV-Adult group compared with Ndufs6^gt/gt^ controls. Specifically, none of the AAV-Adult or Ndufs6^gt/gt^ mice could run for 800 s on the treadmill (Fig. 5C). In brief, AAV9-hNdufs6 gene therapy did not effectively reverse the severe cardiac dysfunction in adult Ndufs6^gt/gt^ mice, which was supported by histological evidence. Cardiomegaly was identified in AAV-Adult mice with an increase in LVEDD (Fig. 5D) and HW/BW ratio (Fig. 5E). Besides, fibrosis in cardiac tissues (Fig. 5F) and cardiomyocyte apoptosis (Fig. 5G) were not relieved after AAV9-hNdufs6 treatment, accompanied with high ROS production (Fig. S3A), severe mitochondrial damage (Fig. S3B) and low CI activity (Fig. S3C), which could not significantly prolong the lifespan of Ndufs6^gt/gt^ mice (Fig. S3D). These findings suggested that AAV9-hNdufs6 therapy could slightly reverse cardiomegaly and improve cardiac function at rest in adult Ndufs6^gt/gt^ mice, but was inadequate to reverse cardiac dysfunction under exercise tolerance.

### Enhanced Kupffer cells (KCs) uptake of AAV may limit the efficacy of gene therapy on MCM in adult mice

**Fig. 6:**
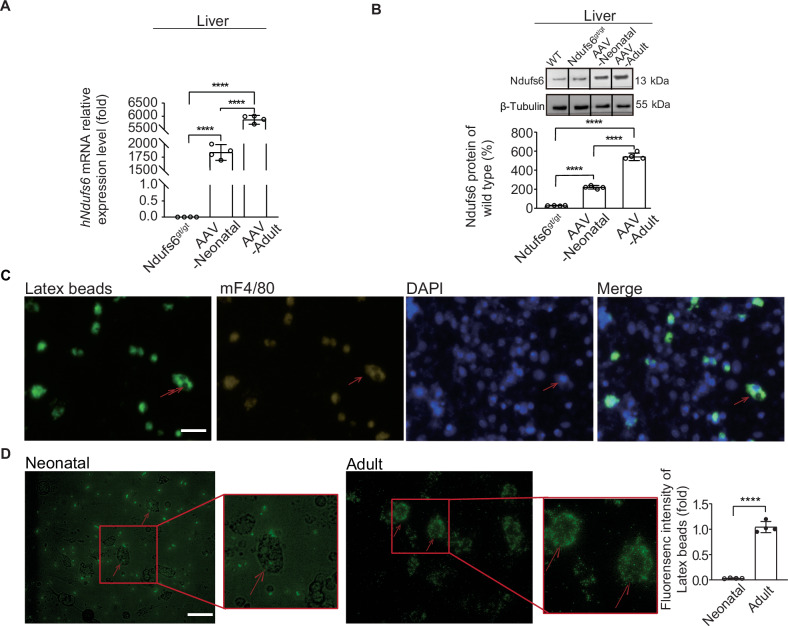
The impact of phagocytosis activity of KCs on the effect of AAV gene therapy. **A** The relative expression of *hNdufs6* mRNA in mice liver of Ndufs6^gt/gt^, AAV-Neonatal and AAV-Adult groups at 3 months post gene therapy (*n* = 4). **B** Relative protein expression of NDUFS6 in mice liver was detected in Ndufs6^gt/gt^, AAV-Neonatal and AAV-Adult groups by Western blot. The quantification results were calculated following two steps by Image J: 1) gray scale for each sample was normalized to GAPDH (WT-1 = WT gray scale/GAPDH gray scale; Ndufs6^gt/gt^-1 = Ndufs6^gt/gt^ gray scale/GAPDH gray scale; AAV-Neonatal-1 = AAV-Neonatal gray scale/GAPDH gray scale; AAV-Adult-1 = AAV-Adult gray scale/GAPDH gray scale); 2) then Ndufts6^gt/gt^-1, AAV-Neonatal-1 and AAV-Adult-1 are expressed as percentage of WT levels. (*n* = 4). **C** Immunofluorescence analysis of macrophages displaying DAPI, mF4/80, and Latex beads. The DAPI staining (blue) marks the cell nuclei, mF4/80 staining (red) identifies the macrophage surface receptor, and green fluorescence highlights the phagocytosed Latex beads within the cells. The merged image shows the co-localization of mF4/80 with the Latex beads, confirming the phagocytic activity of mF4/80-positive cells. Scale bar = 20 μm. **D** Quantification of fluorescence intensity and representative photos of phagocytic activity of KCs in neonatal (PDN1) mice and Adult (3-month-old) mice at 2 h, respectively. Scale bar = 20 μm.

**Fig. 7:**
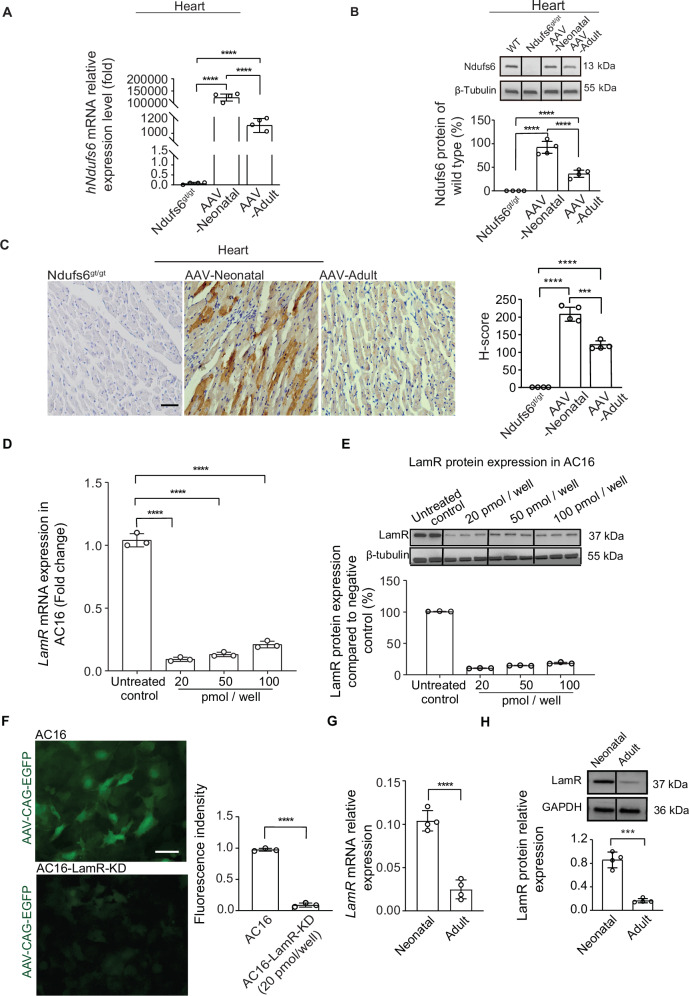
The higher LamR expression in neonatal mice enhances the efficacy of AAV gene therapy in heart. **A** The relative expression of *hNdufs6* mRNA in cardiac tissue of Ndufs6^gt/gt^, AAV-Neonatal and AAV-Adult groups at 3 months post AAV gene therapy (n = 4). **B** Relative protein expression of NDUFS6 in cardiac tissue was detected in Ndufs6^gt/gt^, AAV-Neonatal and AAV-Adult groups by Western blot. The quantification results were calculated following two steps by Image J: 1) gray scale for each sample was normalized to GAPDH (WT-1 = WT gray scale/GAPDH gray scale; Ndufs6^gt/gt^-1 = Ndufs6^gt/gt^ gray scale/GAPDH gray scale; AAV-Neonatal-1 = AAV-Neonatal gray scale/GAPDH gray scale; AAV-Adult-1 = AAV-Adult gray scale/GAPDH gray scale); 2) then Ndufts6^gt/gt^-1, AAV-Neonatal-1 and AAV-Adult-1 are expressed as percentage of WT levels. **C** Representative images and quantification IHC showed Ndufs6 protein expression in the cytoplasm of heart tissues in Ndufs6^gt/gt^, AAV-Neonatal and AAV-Adult mice (*n* = 4). Scale bar=50μm. ***p = 0.0004. **D** *LamR* mRNA expression in AC16 cell line after siRNA treatment at different concentration. (*n* = 3). **E** LamR protein expression in AC16 cell line after siRNA treatment at different concentration. (*n* = 3). **F** Representative images of AC16 cells and AC16-LamR-KD cells 48 h post AAV9-CAG-eGFP infection. Scale bar = 20 um. (*n* = 3). **G** *LamR* mRNA relative expression level in PND0 and 3-month-old mice. (*n* = 4) **H** LamR protein expression and quantitative analysis result on PND0 and 3-Month-old mice (*n* = 4). ***p = 0.0001.

Vector accumulation in liver was indicated a significant factor to impact the therapeutic efficacy of gene therapy on target organs like the heart [25]. The quantity and phagocytosis of KCs increased along with age growth in rats [26]. Based on the different effects of AAV gene therapy between neonatal and adult mouse in our study, further study was conducted to figure out if the different efficacy was relative with their KC function levels. Quantitative analysis of mRNA expression of hNdufs6 in the liver showed that its expression in adult mice was higher than that in the neonatal group (Fig. 6A). Similar trend was observed at protein level with higher protein expression in adult mice (Fig. 6B). Immunostaining was performed to show KCs from liver tissues of adult mice (3-month-old) and neonatal mice (PND 0), which was mouse F4/80 (mF4/80) positive, while their phagocytic activity was tested by phagocytosis of Latex beads over a 24-h period. Phagocytic activity was measured by the fluorescence intensity of cells positive with both mF4/80 and Latex beads (Fig. 6C). The phagocytic capacity analysis showed a higher phagocytosis of KCs (Fig. 6D) in 2 h after the tests. Therefore, a higher retention rate of AAV-hNdufs6 in livers of adult mice could be a root cause for a lower expression rate of hNdufs6 in cardiac tissues (Fig. 7A), which was attributed to a higher phagocytosis of KCs in adult mice.

### Different expression level of Laminin receptor (LamR) on cardiomyocytes between neonatal and adult mice impacted AAV9 infection

The current study demonstrated that AAV9-hNdufs6 effectively prevented the onset of cardiomyopathy in neonatal Ndufs6^gt/gt^ mice but did not significantly improve cardiac dysfunction in adult mice with severe cardiomyopathy. To explore the root causes, we further analyzed the expression of Ndufs6 in mRNA and protein levels with cardiac tissues from different groups of Ndufs6^gt/gt^ mice. As a result, the expression of Ndufs6 increased significantly in mRNA (Fig. 7A) and protein (Fig. 7B) levels for both AAV-Neonatal and AAV-Adult groups. Notably, the expression of Ndufs6 was significantly higher in AAV-Neonatal group than that in the AAV-Adult group (Fig. 7A–C). Additionally, the impact of LamR, a necessary cellular receptor for AAV9 transduction [27], was checked in vitro using a immortalized human cardiomyocyte cell line (AC16). AC16 cells with LamR knocked down (AC16-LamR-KD) were obtained by transfecting with LamR short interfering RNA (siRNA), resulting in approximately 70% knockdown of LamR at mRNA and protein levels (Fig. 7D, E). AC16-LamR-KD cells were then used for AAV9-CAG-eGFP infection at an MOI of 1:100 000 (Fig. 7F). After 72 h, only half of AC16 cells showed green fluorescence, whereas the infection rate of AC16-LamR-KD cells was significantly lower with less and weaker fluorescence intensity, suggesting the AAV9 infection rate was impacted by LamR expression Subsequently, we evaluated the LamR expression level in cardiac tissues from both neonatal (PND 0) and adult mice (3-Month-old). Compared to the neonatal mice, LamR expression was lower in the adult mice at both mRNA and protein levels (Fig. 7G, H). Therefore, a lower LamR expression level in cardiac tissues of adult mice could impact the infection rate of AAV9-hNdufs6, which contributed to a poor therapeutic efficacy of gene therapy for the adult group. LamR could become a potential therapeutic target for gene therapy based on AAV9.

### Good tolerance post AAV9-hNdufs6 administration without liver and renal toxicities

The risks of liver and renal toxicities of AAV-hNdufs6 gene therapy were monitored in our study, which had been reported in clinical trials for other AAV gene therapy [28]. Serum samples were collected from neonatal and adult mice 3 months after AAV-hNdufs6 administration. Alanine transaminase (ALT) and aspartate transaminase (AST) were tested to assess liver function, while serum urea nitrogen (BUN) was tested for renal function. The safety profile was promising since no significant differences were detected in liver and renal function among different groups (Fig. S4A–F). These findings indicated a good tolerance of AAV-hNdufs6 therapy at a dosage of 1e14vg·kg^-1^.

## Discussion

Despite some researches focused on mitochondrial disorders, the therapeutic potential of gene therapy remained elusive for CI deficiency-induced cardiac dysfunction. Here, we present the first study to delineate the therapeutic window for AAV9-hNdufs6 gene therapy based on Ndufs6^gt/gt^ mice, which is a typical model to mimic the clinical phenotype of Ndufs6-deficient mitochondrial cardiomyopathy. Our study also revealed an AMPK-dependent mechanism of AAV9-hNdufs6 therapy which involved ROS generation, mitochondrial fission and cardiomyocyte apoptosis. Last but not least, our study provides the first analysis on the root cause for different efficacy between prophylactic and symptomatic therapies of AAV9-hNdufs6 based on different therapeutic time window in neonatal and adult mice.

The clinical and preclinical studies of AAV gene therapy have been explored for dilated cardiomyopathy (DCM) [29, 30], arrhythmogenic right ventricular cardiomyopathy (ARVC) [31] and hypertrophic cardiomyopathy (HCM) [32]. However, their pathological mechanisms are quite different from mitochondrial CI dysfunction-associated MCM. The pathogenesis of MCM rely on energy metabolism disorders in cardiomyocytes, which is not the primary cause for most of DCM patients. It is necessary to explore the therapeutic potential of AAV gene therapy on MCM with a well-recognized animal model. Therefore, a typical model with gene trap-induced *Ndufs6* deficiency was used in this study to mimic the cardiomyopathy caused by mitochondrial CI dysfunction due to *Ndufs6* mutations. This animal model was reported to simulate the clinical phenotype of CI-deficient mitochondrial cardiomyopathy, and represents severe cardiac dysfunction on left ventricular systolic function, cardiac volume, and cardiac output at four weeks postnatal [11]. So, we employed these Ndufs6^gt/gt^ mice to cast light on the therapeutic potential from laboratory to bedside and present the first preclinical evidence of AAV-hNdufs6 therapy on Ndufs6-deficient MCM. Our results demonstrate robust expression of Ndufs6 in cardiac tissue following systemic administration of AAV9-hNdufs6 in neonatal mice. Notably, AAV9-hNdufs6 treatment effectively halted the deterioration of cardiac function in neonatal mice, with reduced fibrosis, ROS generation, and cardiomyocyte apoptosis compared with the untreated Ndufs6^gt/gt^ mice.

Recent studies have implicated CI deficiency in perturbing mitochondrial dynamics [33, 34], which critically regulates cell function. Enhanced ROS generation can disrupt the balance of mitochondrial fission and fusion [35, 36], leading to enhanced mitochondrial fission in CI deficiency and subsequent cardiac dysfunction [34]. MIEF2 gene mutation promotes mitochondrial fission, suggesting a close interplay between CI and mitochondrial dynamics [37]. In our study, mitochondria in Ndufs6^gt/gt^ mice exhibited smaller and more numerous mitochondria compared to WT mice. Administration of AAV9-hNdufs6 in neonatal mice significantly attenuated mitochondrial fragmentation. Additionally, reduced expression of p-AMPK was observed in Ndufs6^gt/gt^ mice compared with WT mice (Fig. 4C), a critical regulator of mitochondrial dynamics via the AMPK signaling pathway [23, 38, 39]. In our study, we also demonstrated that AAV9-hNdufs6 administration restored p-AMPK expression (Fig. 4C) and mitigated mitochondrial fission by enhancing CI activity (Fig. 4A). Structural abnormalities in mitochondria can induce cell apoptosis by facilitating the release of enzymes and molecules from mitochondria to the cytoplasm [40]. Following AAV9-hNdufs6 treatment, we observed a significant reduction in cardiomyocyte ROS levels, leading to the reversal of mitochondrial pathology and mitigation of cardiomyocyte apoptosis.

Last but not least, superior efficacy of AAV9-hNdufs6 gene therapy was observed for prophylactic therapy in neonatal mice compared with symptomatic therapy in adult mice. Notably, more accumulation of AAV in liver and less anchoring vectors in cardiac tissues were identified as root causes for poor efficacy in adult mice. Consistent with our study, the impact of viral vector accumulation in liver had been reported in clinical trials of AAV-based gene therapy. In that case, the therapeutic efficacy was diminished significantly as the delivery of AAV vectors to target organs was limited due to accumulation in liver. However, the detail mechanism remained unclear. in liver tissues. Therefore, further study was conducted in our study to explore the mechanism. In previous study, KCs were reported to internalize AAV9 particles under basal conditions [41], which were enhanced along with the age growth in rats [26]. In accordance with previous study, higher phagocytosis of KCs in liver was detected in adult mice based on our data (Fig. 6C), which resulted in more accumulation of AAV vectors in liver of adult mice compared with neonatal mice. It was accounted as one of the root causes for a poor efficacy of symptomatic therapy in adult mice.

Another importance factor for the therapeutic efficacy was supposed to be the expression level of LamR, which played a role in the binding and internalization of AAV9 viral particles [42]. Previous research had shown that higher expression level of LamR led to higher AAV9 transduction efficiency in lung of mice [43]. However, it was not clear if the same impact existed in cardiac tissues of Ndufs6^gt/gt^ mice. Therefore, we detected the LamR expression in cardiac tissues between neonatal and adult mice to check its relative with the differences of AAV9 infection efficiency. As a result, a positive correlation was identified between LamR expression level and AAV9 infection efficiency in cardiac tissues (Fig. 7F). Additionally, a lower LamR protein expression level was detected in cardiac tissues from adult mice compared to neonatal mice (Fig. 7G, H), which was suggested a significant impact factor for the therapeutic efficacy. Therefore, the mechanism of different efficacy between prophylactic and symptomatic therapies was suggested relative with higher phagocytosis of KCs in liver and lower expression of LamR in cardiac tissues. The safety of AAV gene therapy was also concerned for liver toxicity due to the vector accumulation in liver, especially at a high dose [44]. Therefore, liver function was monitored in our study based on AST and ALT, and no significant difference was detected between mice with and without gene therapies (Fig. S4A, B, D, E). Similarly, no impact of gene therapy on renal function was detected based on the UREA tests (Fig. S4C, SF). So, the dosage of AAV9-Ndufs6 gene therapy in our study was safe and valuable for a reference to further studies.

Our study contributes novel insights into gene therapy for CI dysfunction-induced MCM. AAV-based gene therapy administered during the neonatal stage effectively prevents the onset of cardiomyopathy and restores cardiac function. However, application of gene therapy to adult mice with established cardiac impairment yields limited efficacy and is unable to restore cardiac function from heart failure, underscoring the importance of early intervention in future clinical application.

Despite its significant findings, this study has several limitations. Firstly, the dosage of AAV9-hNdufs6 was determined based on previous studies, necessitating further exploration to ascertain dose-dependency. Secondly, further extensive long-term studies are warranted to fully elucidate the sustained therapeutic effects of AAV9-hNdufs6 in cardiomyopathy with Ndufs6 deficiency. Thirdly, the overall immunogenicity profile of AAV9-hNdufs6 was not assessed comprehensively.

## Conclusions

Our study underscores the efficacy and safety, as well as the therapeutic timeframe of AAV9-hNdufs6 therapy on Ndufs6-deficient cardiomyopathy. Prophylactic therapy was proved effective to prevent cardiac dysfunction and prolong lifespan in neonatal Ndufs6-deficient mice. The underlying mechanism involves downregulation of ROS generation to mitigate mitochondrial fission and cardiomyocyte apoptosis via the AMPK signaling pathway. However, the efficacy of symptomatic treatments was not sufficient in adult Ndufs6-deficient mice, which attributed to a higher vector retention rate in liver and lower vector infection rate in cardiomyocytes. Based on the promising efficacy and safety profiles in the current study, AAV9-Ndufs6 gene therapy proves to be a viable strategy for Ndufs6 deficiency-induced MCM. We need to improve the AAV infect efficiency in heart and reduce its accumulation in the liver. Further clinical study is needed to identify the patient population sensitive to correction via overexpression of Ndufs6.

## Materials and methods

### Preparation of AAV9-hNdufs6 virus

The CAG promoter, a hybrid of cytomegalovirus (CMV) enhancer and the chicken beta-actin promoter, was compared with the chicken troponin (cTNT) promoter in our study, which is known for its specific expression in cardiomyocytes. Both AAV9-CAG-eGFP and AAV9-cTNT-eGFP were predominantly trapped in the liver without significant difference in expression levels in cardiac tissues (results not showed). Consequently, we selected the CAG promoter for our gene therapy due to its robust ability to promote protein expression in mammalian cells. The plasmid AAV9-CAG-eGFP, obtained from Partick Aubourg/Mofrgane, served as the basis for reconstructing AAV9-CAG-hNdufs6, wherein eGFP was replaced with the full-length human Ndufs6 cDNA. AAV was produced using a previously published protocol [45]. Briefly, HEK293T cells were transfected with three plasmids containing AAV9-Rep/Cap, the target gene flanked by ITRs, and essential adenovirus helper genes. After 96 h, cells were lysed, and both the medium and cell lysate were collected. AAV particles were purified using POROS Capture Selet AAV9 Affinity Resin (GIBCO, A27354) following the manufacturer’s protocol and stored at -80°C. AAV titration was performed according to the protocol on Addgene website (https://www.addgene.org/protocols/aav-titration-qpcr-using-sybr-green-technology/) using two primers flanking the ITR region used for qPCR titration (ITR F: 5’-GGAACCCCTAGTGATGGAGT; ITR R: 5’-CGGCCTCAGTGAGCGA).

### Animal model

Ethical approval for the study was granted by the Animal Ethics Committee on the Use of Live Animals in Teaching and Research (CULATR). All animal procedures were conducted at Huateng Biology Company in accordance with relevant guidelines and regulations (IACUC No. HTSW220112). The Ndufs6^gt/gt^ mice [46], obtained from Dr. Thorburn’s lab, were created using a gene-trap method to knockout the Ndufs6 subunit in cardiac tissue while retaining small amounts of WT *Ndufs6* mRNA in other tissues. Genotyping was performed using primers A, B and C, which produced products of 489 bp (WT allele) and 240 bp (KO allele), respectively (Fig. S1A; Primer A, GAGTGAGGACGAGGAGAGTTG; Primer B, CCATGGCCTTCTAAATTCAGGT; Primer C, AAGTGGTGGCCTAACTACGG). Genomic DNA from mouse tail samples was isolated by 100 μl Solution I (25 mM NaOH, 0.2 mM EDTA) at 95°C for 45 min, followed by the addition of 100 μl Solution II (40 mM Tris-HCl, adjusted to pH5.5 by HCl) after cooling to room temperature (RT). The resulting mixture was directly used for PCR with Green Taq Mix (Vazyme, P131) following the thermo-program: 95 °C for 3 min.; 32 cycles of 95 °C for 15 s, 60 °C for 15 min, 72 °C for 30 s; and a final extension step of 72 °C for 5 min. Amplicon sizes were analyzed by electrophoresis using 1.0% agarose gel with Super red/gel red (Biosharp, BS354B).

### Animal groups

Previous study has shown that Ndufs6^gt/gt^ mice develop more severe cardiac dysfunction in males than in females due to the absence of estrogen’s protective effect on cardiac function [34]. Consequently, only male mice were utilized in our study to avoid such bias. Ndufs6^gt/gt^ neonatal mice (AAV-Neonatal group) received prophylactic therapy of AAV9-hNdufs6 via tail intravenous injection at PND0, while adult mice (AAV-Adult group) received AAV9-hNdufs6 administration after the onset of cardiomyopathy at 3 months postnatal. AAV9-hNdufs6 was given at a dosage of 1e14 vg·kg^-1^ for both groups. WT mice and untreated Ndufs6^gt/gt^ mice were used as parallel controls. Cardiac function assessment was performed based on echocardiography and treadmill testing 3 months after the treatment. For AAV-adult group, we select 6 mice for AAV therapy group and the parallel control. Since neonatal mice are more vulnerable and subject to higher variability in responses, 7 mice were chosen for AAV-neonatal group. For the survival rate experiments, with 15 mice per group, we considered that survival - related studies often involve multiple confounding factors and require a larger sample size to accurately capture the differences in survival probabilities.

### Cardiac echocardiography assessment

Cardiac function at rest was evaluated via echocardiography at Guangdong Provincial People’s Hospital. In brief, mice were taken into the procedure room to acclimate for at least 30 min before the assessment, then were anesthetized with 1–2% isoflurane mixed with 100% oxygen. Under gas anesthesia, the mice received echocardiography exams in a supine position on a heating pad to maintain body temperature at 37 °C. Echocardiographic parameters were measured using a high-resolution Micro-Ultrasound system (Vevo 770, Visual Sonics Inc.) equipped with a 25-MHz linear transducer to obtain parasternal long-axis and short-axis views of the left ventricle. Record M-mode images to assess cardiac function and structure. Data were collected at least for three cardiac cycles in each measurement and analyzed using VevoLab software (VisualSonics). EF% and FS% of left ventricle were analyzed for cardiac function assessments. LVEDD is used to reflect changes in left ventricular size, providing data for the identification of cardiac enlargement or hypertrophy. Finally, the mice recovered from anesthesia by gradually reducing the isoflurane concentration, and monitored in a warm, clean cage for revitalization.

### Treadmill tolerance test

Exercise tolerance was assessed by mice running time in treadmill exercise based on previously published protocols [22, 47]. The treadmill test began at a speed of 10 m min^−1^ for 1 min, followed by an increase to 15 m min^−1^ for the next 2 min. Subsequently, the speed was raised to 20 m min^−1^ min and incline was increased to 15°, with the shock grid configured at 5 Hz. Each mouse was placed in a separate lane on the treadmill, and the corresponding grid was immediately activated. The timing of the treadmill tests was synchronized, with careful monitoring for the tolerance of all mice throughout the testing period. The mice with poor tolerance were promptly removed from the treadmill if they remained in the fatigue zone for five consecutive seconds. Each mouse underwent testing and observation for 1800 s.

### Histological analysis

After completing cardiac function assessments, all mice were sacrificed to harvest cardiac tissues, and calculated the HW/BW ratio based on the weight of heart and body of mice. Then the cardiac tissues were embedded in paraffin and sectioned into slides with sickness as 5 μm. Hematoxylin and eosin (H&E) staining and Masson’s trichrome staining were performed according to the manufacturer’s instructions (Servicebio, G1005 and G1006) to assess fibrosis in cross-sectional areas (CSA) of the left ventricle. For paraffin sections, deparaffinize sections sequentially (xylene: 10 min; fresh xylene: 10 min; anhydrous ethanol: 5 min; fresh anhydrous ethanol: 5 min; 90% ethanol: 5 min; 75% ethanol: 5 min; wash in tap water). For frozen sections, allow sections stored at −20 °C to stand for 5–10 min to return to RT. Fixed tissue sections with 4% paraformaldehyde (PFA) for 15 min at RT, then follow the H&E or Masson’s trichrome staining.

H&E staining was performed according to following procedure. Sections were put in hematoxylin staining solution for 3–5 min, then washed with tap water. Differentiation was performed by rinsing in hematoxylin differentiation solution for 2–5 s under running tap water, then rinsed in hematoxylin back-to-blue solution for 2–5 s, followed by rinse well with running tap water. Eosin staining began with dehydrating in 85% and 95% gradient ethanol for 5 min each, then stained in alcoholic eosin for 5 min. The sections were dehydrated twice with absolute ethanol for 5 min each, then for another 5 min with fresh absolute ethanol. Cleared sections in xylene for 5 min, then for another 5 min with fresh xylene before adding a drop of neutral gum mounting medium.

Masson’s trichrome staining was performed according to the manufacturer’s instructions. Briefly, the tissue sections were sequentially stained with the following solutions: 2.5% potassium dichromate, Weigert’s iron hematoxylin dye solution (mixed in equal volume), Ponceau acid fuchsin, 1% phosphomolybdic acid solution, and 2.5% aniline blue solution. After staining with Masson’s trichrome, collagen fibers appear sky blue to dark blue, muscle fibers, cytoplasm, cellulose, and keratin appear red to purplish-red, and red blood cells appear light red.

Fibrosis in cardiac tissues was determined by the ratio of fibrotic area to the total tissue area in calculating fields. Five random fields were selected on each slide for analysis using StrataQuest (Version 7.1, TissueGnostics, Austria) with application of a threshold color plug-in for image processing.

### Immunohistochemistry and immunofluorescence staining

Immunofluorescence staining (IF) was used to visualize the presence of AAV in frozen liver tissue sections, following the manufacturer’s protocol available on the website (https://www.abcam.com/protocols/immunocytochemistry-immunofluorescence-protocol). Briefly, 8 μm frozen liver sections were fixed with 4.0% PFA for 10 min at RT, followed by incubation in 0.5% Triton X-100 for 10 min at RT. After blocking by 1.0% BSA for 1 h, liver sections were treated with AAV antibody (Abcam, ab45482) working solution (1:200) at 4 °C overnight. Subsequently, sections were incubated with Anti-Rabbit IgG Secondary Antibody (SAB, L3016) (1:200) at RT for 1 h. Finally, liver sections were incubated with 0.1 μg·ml^-1^ Hoechest for 1 min. Slides were observed under a Leica DMi8 microscope, and quantification data were analyzed using Image J, with 5 random fields for each slide.

Immunohistochemistry (IHC) was applied to observe the presence of Ndufs6 protein in cardiac tissue sections. IHC was performed according to the manufacturer’s direction on the website (https://www.abcam.com/protocols/immunocytochemistry-immunofluorescence-protocol). Briefly, paraffin-embedded cardiac tissue was labeled with Ndufs6 antibody at 1:500 (Abcam, ab195807) for 4 h at 4 °C. Then slides were incubated with secondary antibody (goat Anti-Rabbit IgG H&L, HRP, SAB, L3012) at 1:500 for 1 h at RT. Results were observed under a Leica DMi8 microscope. Quantification data were analyzed using Image J with 5 random fields for each slide. The immunohistochemical staining intensity was assessed using the H-score method. Staining intensity was classified into four categories: 0 (no staining), 1+ (weak), 2+ (moderate), and 3+ (strong). For each category, the percentage of positively stained cells was estimated. The H-score was calculated using the formula: H-score = ∑(P_i_×I_i_), where P_i_ represents the percentage of cells stained at each intensity, and I_i_ is the corresponding intensity score. The final H-score ranges from 0 to 300, with higher scores indicating stronger overall staining.

### Transmission electron microscopy

Cardiac tissue samples were taken from the left ventricular wall for all groups of mice after anesthetization, and then chopped into 1 cubic millimeter of blocks. The tissue blocks were immediately placed into pre-cooled fixative medium and then sent to Servicebio Biotechnology Co., Ltd. (Wuhan) for TEM exams (HITACHI, HT7700, Japan). Mitochondrial ultrastructure was detected and fragmentation was calculated by the ratio of fragmented mitochondria to the total number of mitochondria in each field of view, termed the mitochondrial fragmentation rate. Cirstea damage was determined by calculating the cristae area per mitochondrial area as a percentage using the formula: (cristae area / mitochondrial area) × 100%. Based on the resulting percentage, classify the findings into different classes: Class I: 100% - 85%; Class II: 85% - 50%; Class III: 50% - 25%; Class IV: less than 25%. Six fields of view were analyzed for each sample.

### DHE staining

The detection of superoxide anion (O^2-^) oxygen radicals in cardiac tissue was performed to determine ROS production using DHE staining. Frozen sections of cardiac tissue were incubated with DHE (2e6 mol L^−1^) at RT for 30 min. DHE fluorescence was captured by a fluorescent microscope with a 585 nm filter. The fluorescence intensity was analyzed using Strata Quest (Version 7.1, Tissue Gnostics, Austria), which had a positive correlation with ROS production.

### TUNEL staining

Apoptotic cells were measured by terminal deoxynucleotidyl transferase dUTP nick end-labeling TUNEL staining (MCE, HY-K1078). Generally, dewaxed and rehydrated the paraffin-embedded cardiac tissue sections, then treated tissue sections with 1 μg ml^−1^ Proteinase K in a 10 mM Tris solution for 15 min at RT. Subsequently, all samples were incubated with 50 μl of the TUNEL reaction mixture in a humid chamber for 1 h at 37 °C. Following three washes with PBS, the sections were mounted with DAPI and examined under a fluorescent microscope. To calculate the apoptosis rate, images were captured using a fluorescence microscope. The total number of TUNEL-positive (apoptotic) nuclei and DAPI-positive (total) nuclei were counted in randomly selected fields. The apoptosis rate was calculated as follows: Apoptosis Rate = TUNEL-positive nuclei / Total nuclei (DAPI-positive) ×100%.

### Western blot

Total protein was extracted from different samples, which concentration was determined using the bicinchoninic acid (BCA) protein assay kit (Beyotime, P0011). Western blot was employed to identify the expression level of target protein. In brief, proteins were separated on a 12.0% polyacrylamide gel and then transferred onto a 0.22 μm PVDF membrane (Millipore, ISEQ00010). Next, the membranes were blocked with 5.0% fat-free milk in TBST and incubated overnight at 4°C with specific antibodies (Abcam ab195808, ab128915, Immunoway, YM3030; ServiceBio, GB114323, GB112669, GB115659; CST, # 4494). Subsequently, all membranes were incubated with horseradish peroxide-conjugated anti-rabbit or anti-mouse secondary antibody (SAB, L3012 or L3032) at 37 °C for 1 h. After excess secondary antibody was removed by additional washes, chemiluminescent substrate was added to the membranes, which was exposed to a digital imaging system (Bio-Rad, ChemiDoc XRS+) to visualize the protein bands. The expression levels of target proteins were analyzed by ImageJ software (NIH, Bethesda, MD, US) based on the intensity of the protein bands against a loading control for normalization.

### RNA extraction and q-RT-PCR

Total RNA was extracted using TRIzol reagent (ThermoFisher, 15596026) according to the manufacturer’s guidelines. The RNA was treated with DNase I (ThermoFisher,12185010) and subsequently stored at -80°C. For reverse transcription, 1 µg of DNase-pretreated RNA was used as input with the PrimeScript RT reagent Kit with gDNA Eraser (TAKARA, RR047A). Gene expression was analyzed by q-PCR using TB Green Premix Ex Taq II (TAKARA, RR820B) and QuantStudio 6 Real-Time PCR Systems. Primers Ndufs6_mQP_F and R (Ndufs6_mQP_F: GGGTTTCGGGGTTCAAGTGT; Ndufs6_mQP_R: GGTGCTCCACCTCATTCACA) were employed for amplifying mouse Ndufs6 cDNA. While primers Ndufs6_hQP_F and R (Ndufs6_hQP_F: GAGACTCGGGTGATAGCGTG; Ndufs6_hQP_R: GTGGTGCTGTCTGAACTGGA) were designed for human Ndufs6 cDNA sequence amplification. Primers hLamR_F and R (hLamR_F: AACACAGATTCTCCCCTGCG; hLamR_R: CGGGAGATAGTACCTCGCAT) were employed for amplifying human LamR cDNA.

### Mitochondrial CI activity assay

Mitochondrial CI activity was measured with cardiac tissues according to the manufacturer’s instructions (Solarbio, BC0515). CI catalyze the conversion of NADH to NAD^+^, and its enzymatic activity can be determined by measuring the rate of NADH oxidation, which is monitored by the decrease in absorbance at 340 nm. In brief, cardiac tissue was collected on ice, homogenized, and followed by centrifugation at 600 × *g* for 10 min and then at 11,000 × *g* for 15 min at 4 °C. The resulting sediment was treated with solution I and II followed by ultrasonication. The activity of CI was calculated based on detection at OD 340 nm by a microplate reader (Thermo Scientific Multiskan GO).

### RNA Sequencing and Data Analysis

Total RNA was extracted from cardiac tissues, which concentration and purity were measured using a spectrometer (Nanodrop 2000). The integrity of the RNA was assessed by agarose gel electrophoresis, and the RNA Quality Number (RQN) value was determined using a fragment analyzer (Agilent 5300). The obtained mRNA was reverse-transcribed to synthesize stable double-stranded cDNA, and end repair mix was used to create blunt ends before adapter ligation. The cDNA products were then purified, size-selected, and used as templates for PCR amplification to construct the library. Qualified libraries were sequenced on the Illumina Novaseq X Plus platform. Gene expression analysis was conducted based on Fragments Per Kilobase of transcript per Million mapped reads (FPKM) data for all isoforms. Differential gene expression analysis between samples was performed using DESeq2 software, identifying differentially expressed genes (DEGs) with a selection criterion of *P* value < 0.05 and log_2_ fold change (log_2_FC) ≥ 1. Functional enrichment analysis of DEGs was conducted using the KEGG and GO database. The code used for data analysis is available at https://github.com/QiangSu/code_for_Ndufs6_mus. The raw sequence data generated from mRNA sequencing in this study have been stored in the Sequence Read Archive (SRA) at NCBI, with the accession number PRJNA1161214 at https://www.ncbi.nlm.nih.gov/sra/PRJNA1161214.

### Serum biochemical tests

Blood samples were collected from the orbital venous plexus of mice, and allowed to stand at RT for 30 min before centrifuging at 3000 rpm for 15 min. Serum was obtained and stored at 4 °C. AST, ALT, and UREA were detected using a blood biochemical analyzer (HITACHI3100).

### RNA interference with LamR

LamR is a membrane protein belonging to the inner nuclear membrane protein family, which is closely associated with the structure and function of the cell nucleus. It acts as a receptor for AAV9, facilitating the entry of viral particles into the cells. A short interfering RNA (siRNA) and a control siRNA (Genecreate Biological Engineering CO.) were transfected into AC16 human cardiomyocyte cell line (ATCC, CRL-3568) with lipofect transfection reagent (Lipofectamine 3000, Invitrogen, L3000001) at different concentration follow the instruction. Knockdown efficiency was confirmed based on mRNA expression at 48 h post transfection and protein quantification analysis at 72 h post transfection. AC16 cells were tested with mycoplasma contamination (Biori, #BP-QN01-100), the test result turned out to be negative as of [01052024], demonstrating that this cell line was no mycoplasma contamination.

### In vitro infection by AAV-CAG-eGFP

The AC16 and AC16-LamR-KD were exposed to AAV-CAG-eGFP doses ranging in multiplicity of infection (MOI) from 2000 to 100,000. Cells were plated on 96-well plates with 5000 cells per well. The virus was added to the medium 24 h after plating.

### Mice KCs isolation

KCs, specialized macrophages located in the liver, are essential for the organ’s innate immune defense. The KCs were isolated from PND0 and three-month-old C57/BL6 mice, which were purchased from CarrotBio (Room A28, No. 6, Yinggang Community, Huangpu District, Guangzhou, China). A modified method for KCs isolation was used [48]. The newborn mice were anesthetized by positioning them on an ice pack, then the liver was excised and immediately placed in ice-cold PBS. The adult mice received anesthesia through intraperitoneal injection of pentobarbital, the cardiac perfusion was performed via the portal vein with ice-cold PBS to eliminate red blood cells. The liver was minced and subjected to treatment with 0.1% collagenase IV (Huayun HYJ524) and then incubated at 37°C in a water bath. The homogenized tissue was filtered through a 70 µm strainer to remove remaining undigested chunks, followed by centrifugation at 300 × *g* at 4°C for 5 min. The cell sediments were resuspended in 10 ml RPMI 1640 and then subjected to centrifugation at 50 × *g* for 5 min at 4 °C to eliminate liver parenchymal cells. Repeated this procedure twice. The supernatant from the final centrifugation was collected and centrifuged at 300 × *g* for 5 min at 4 °C again, then the cell pellets were retained. The collected cells should include KCs, sinusoidal endothelial cells and satellite cells. For the purification, seeded cells at 1-3e7 cells per well in a 6-well plate using RPMI1640 medium supplemented with 10% fetal bovine serum and 100 U ml^-1^ Penicillin/Streptomycin. After 2-h culture, non-adherent cells were discarded, leaving behind the adherent KCs.

### Phagocytic assay

Latex beads (Sigma, L4655) were diluted at 1:1000 in PBS, and added 1 μl into the isolated KCs in 12-well plate. Fluorescence intensity was detected after incubation for 2 h, which had positive correlation with the phagocytic capacity of KCs. And 24 h incubation for KCs identification by the following immunofluorescence staining.

### KCs identification by immunofluorescence

Isolated cells were fixed by 2% paraformaldehyde (PFA) for 10 min at RT. Then blocked by 0.5% BSA in 0.1% PBS-Tween for 15 min at RT. Then cells were incubated with mF4/80 monoclonal antibody (1:200) (eBioscienc, #13-4801-82) at 4 °C overnight. Cells were washed by 0.1% PBST for 3 times. Then a goat anti-rat secondary antibody (1:400) (ThermoFisher scientific, # A-11007) was incubated with cells in RT for 1 h. Washed cells with 0.1% PBS Tween for 3 times. Added DAPI (1:1000) to stain nucleus at RT for 5 min.

### Body weight and lifespan observation

Body weight was monitored every 5 weeks from 5 weeks to 25 weeks of age, with measurements taken using a calibrated digital scale to ensure accuracy. The data were recorded to track changes over time. Additionally, the lifespan of each mouse was documented from weaning until 240 days postnatal, allowing us to evaluate the long-term effects of the gene therapy intervention.

### Statistical analysis

Statistical analysis was performed using GraphPad Prism 8 (GraphPad Software Inc., USA). Data were presented as mean (±standard deviation). Survival analysis was performed using Kaplan-Meier survival curves and compared by log-rank tests between groups. Differences between groups were analyzed by unpaired t-test. Significance among multiple experimental groups versus a control group was determined by One-way ANOVA analysis with Dunnett’s multiple comparisons test. One-way ANOVA with Tukey’s test is used when comparing the means of three or more independent groups to determine if there are any statistically significant differences among them. A *P* value < 0.05 was considered statistically significant. ****p < 0.0001.

## Supplementary information

Figure S1. Genotyping and Ndufs6 mRNA expression and differential gene expression in transcriptomics of Ndufs6gt/gt mice.

Figure S2. Biodistribution of AAV-eGFP in WT adult mice.

Figure S3. AAV-hNdufs6 prevent heart dilation and restore mitochondrial function in neonatal mice.

Figure S4. Serum indicators of liver and kidney function after AAV gene therapy.

Supplementary legends

Supplemental figures of western blot.

## Data Availability

The datasets generated during and analysed during the current study are available from the corresponding author on reasonable request.
