# Supplementary figures and images for: Gene therapy prevents onset of mitochondrial cardiomyopathy in neonatal mice with Ndufs6 deficiency
Source: Cell Death Discov. 2025 May 22;11:249. doi: 10.1038/s41420-025-02524-7 (PMC12095822; doi:10.1038/s41420-025-02524-7)

A

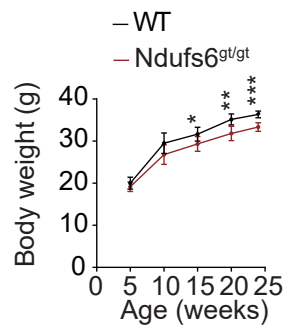

B

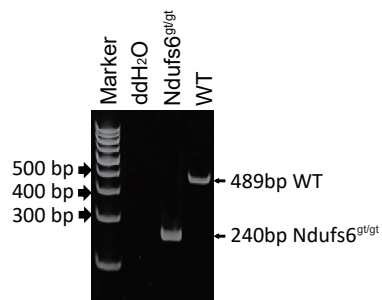

D

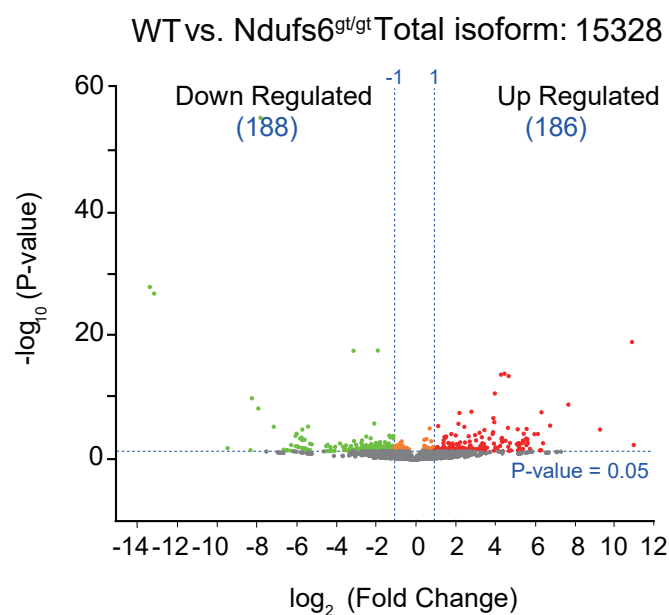

C

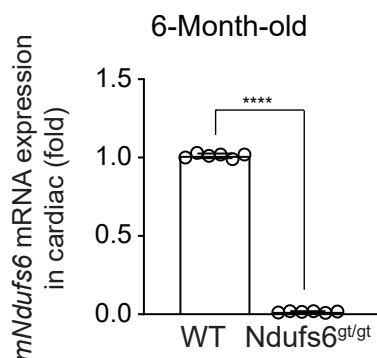

E

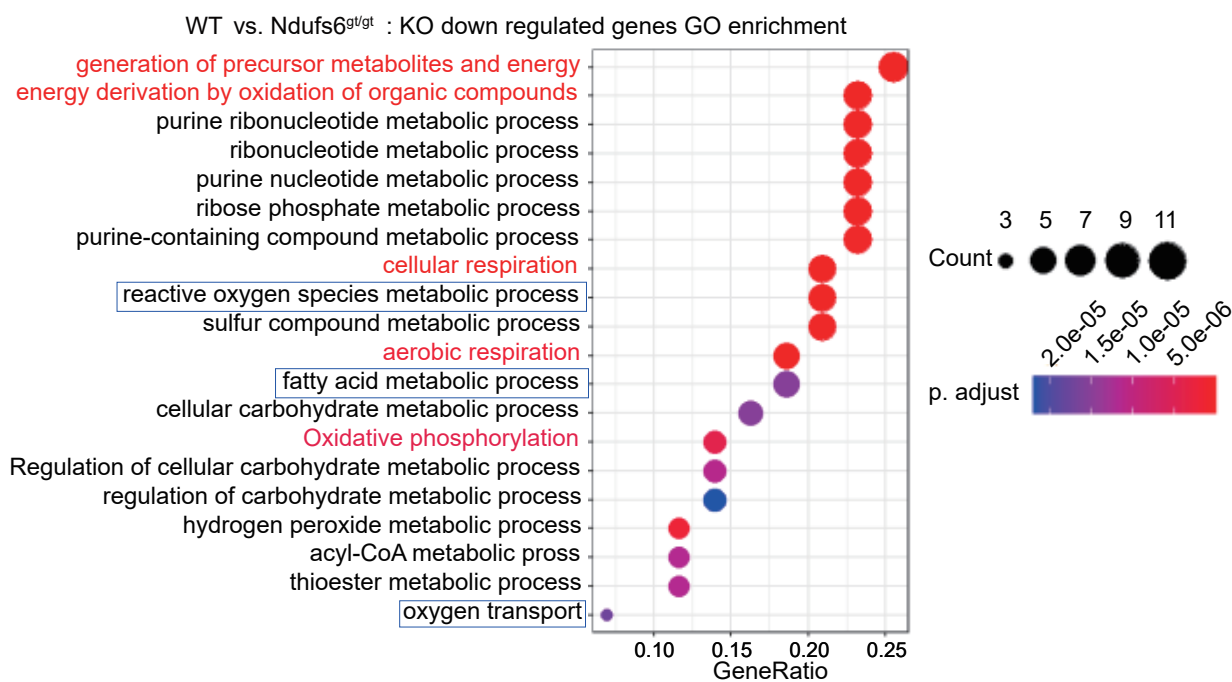

F

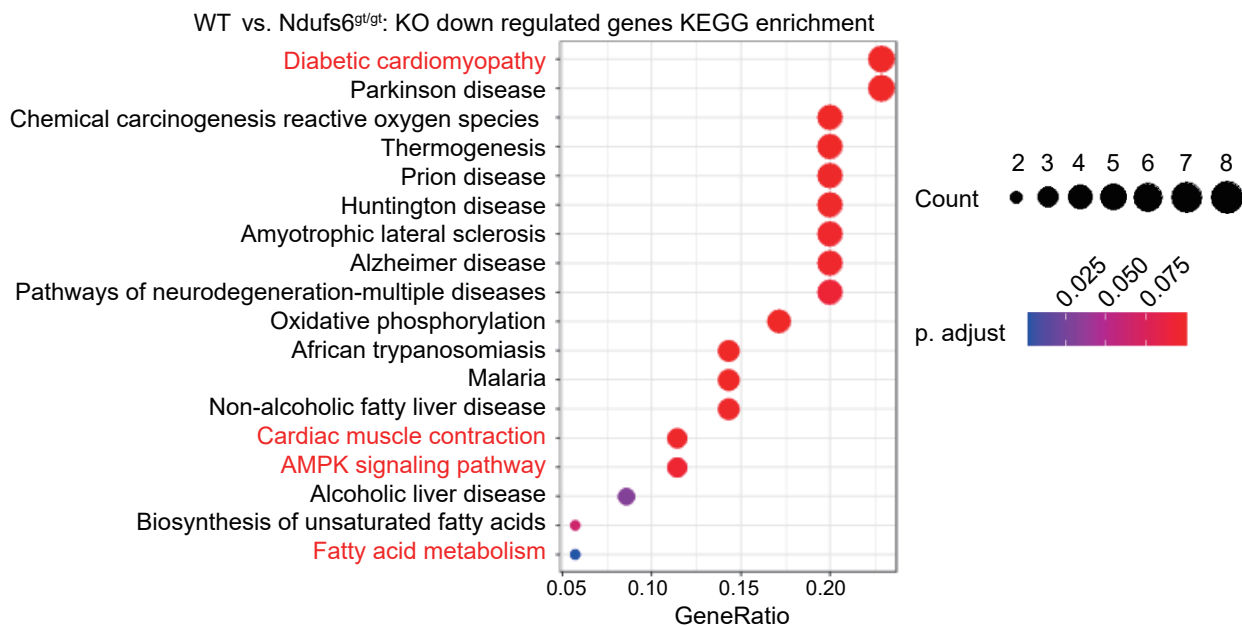

Supplement: Supplementary file 1 — Figure S1. Genotyping and Ndufs6 mRNA expression and differential gene expression in transcriptomics of Ndufs6gt/gt mice. [file 41420_2025_2524_MOESM1_ESM.pdf]

**A**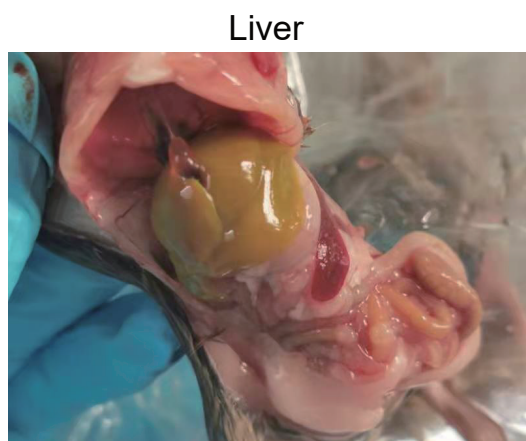**C**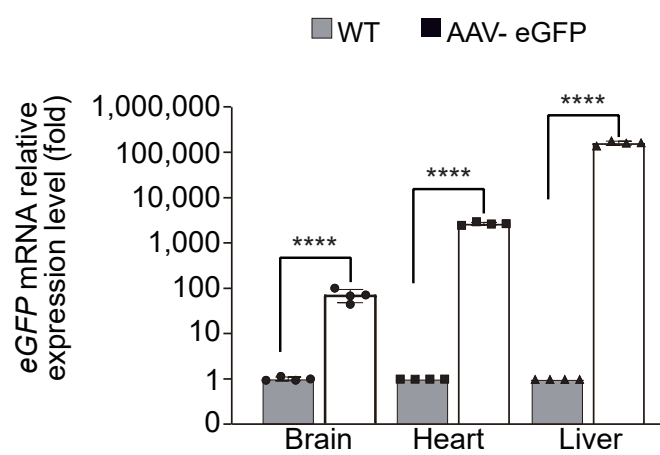**B**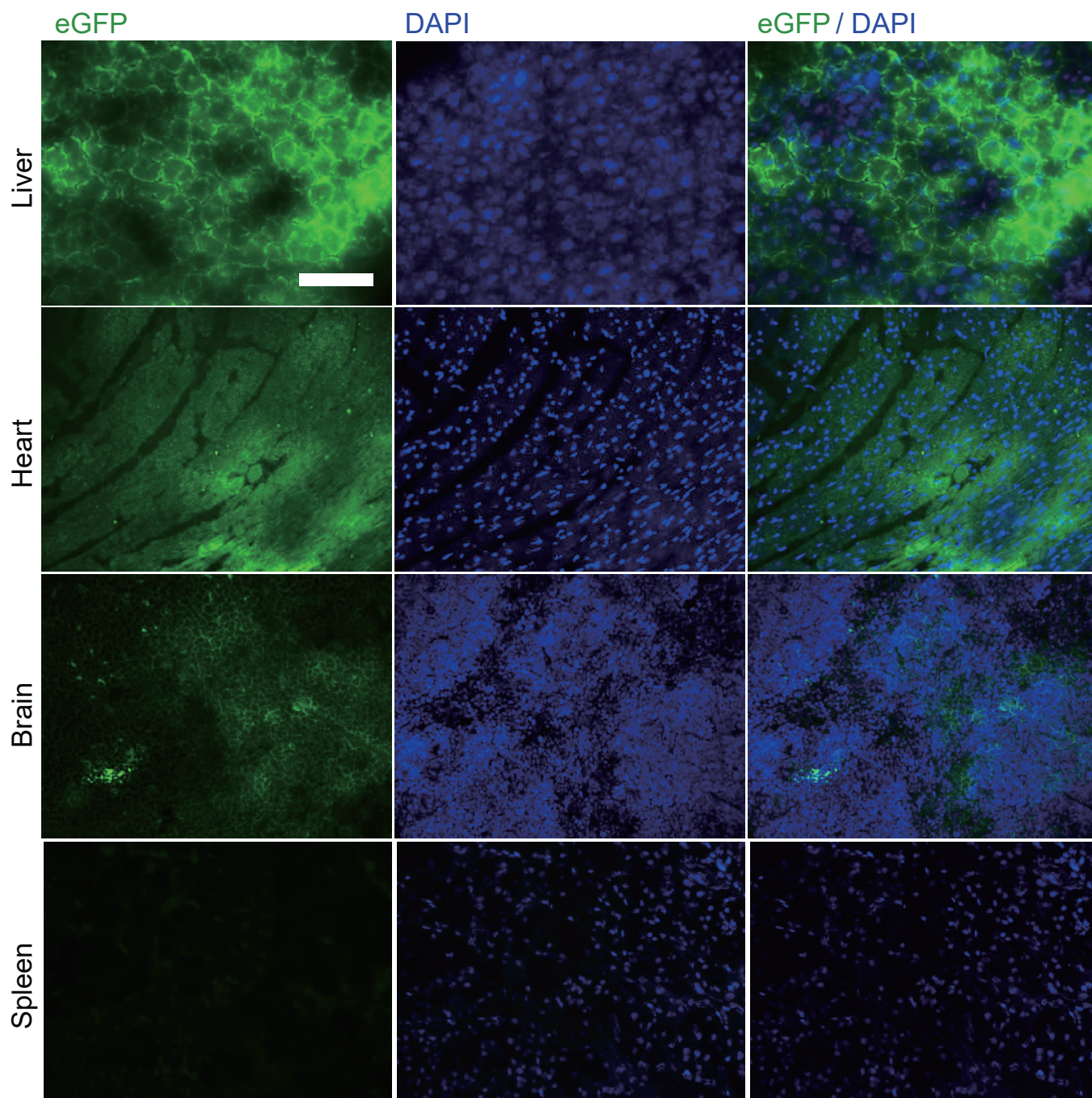

Supplement: Supplementary file 2 — Figure S2. Biodistribution of AAV-eGFP in WT adult mice. [file 41420_2025_2524_MOESM2_ESM.pdf]

A

6-Month-Old

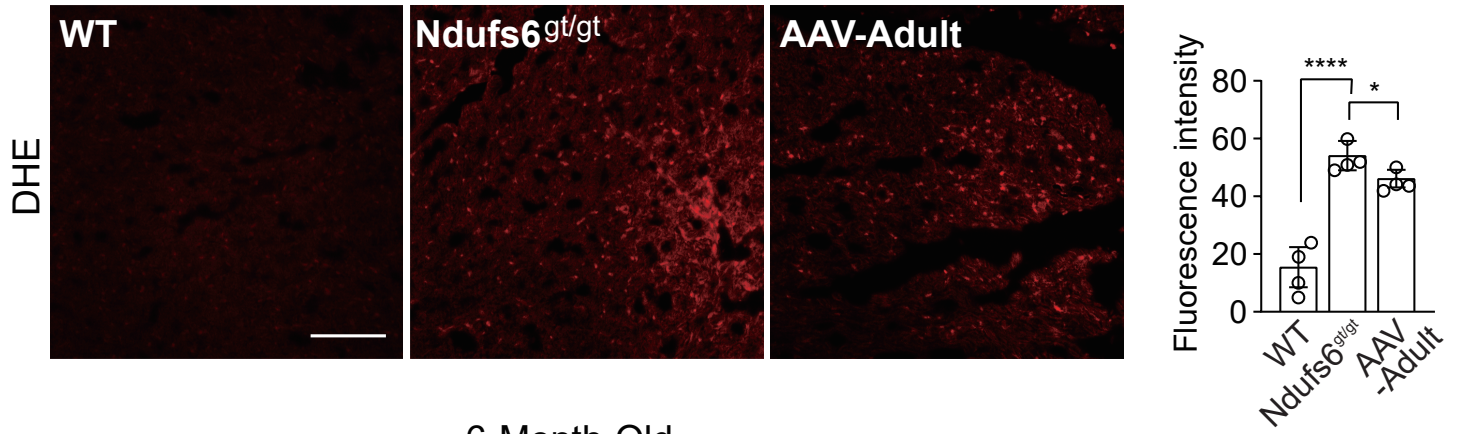

B

6-Month-Old

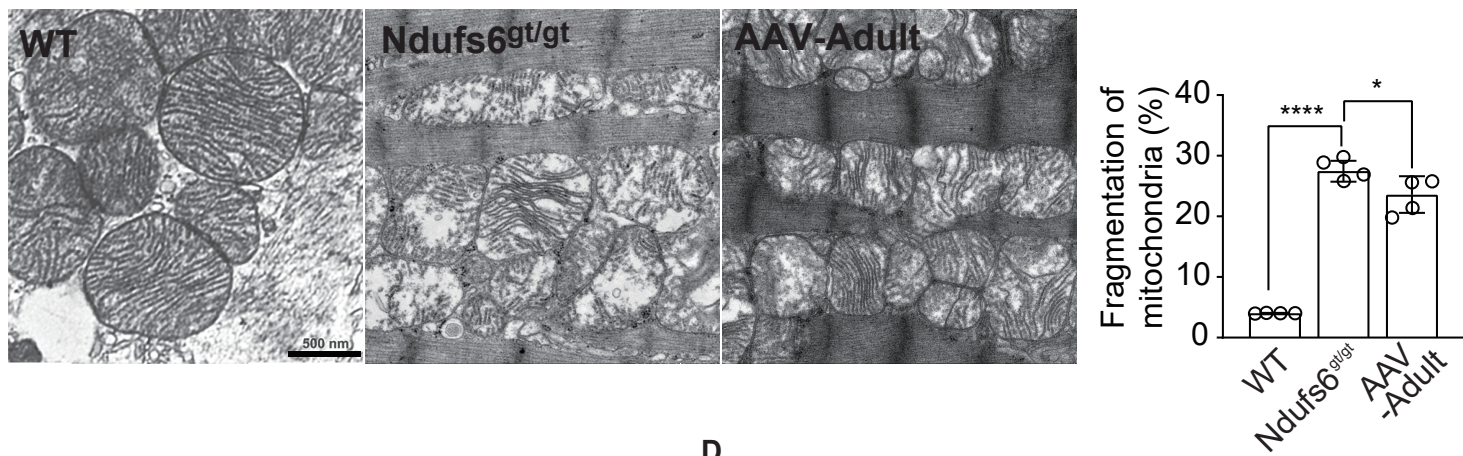

C

6-Month-Old

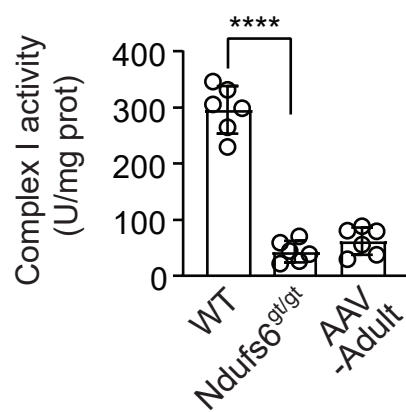

D

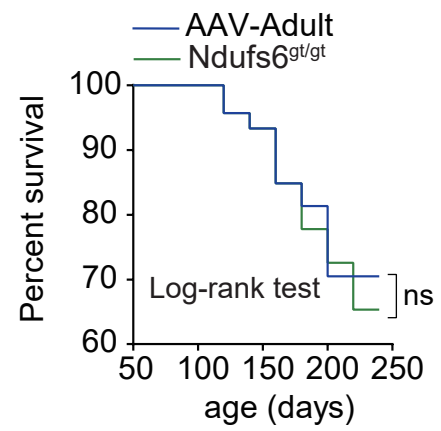

Supplement: Supplementary file 3 — Figure S3. AAV-hNdufs6 prevent heart dilation and restore mitochondrial function in neonatal mice. [file 41420_2025_2524_MOESM3_ESM.pdf]

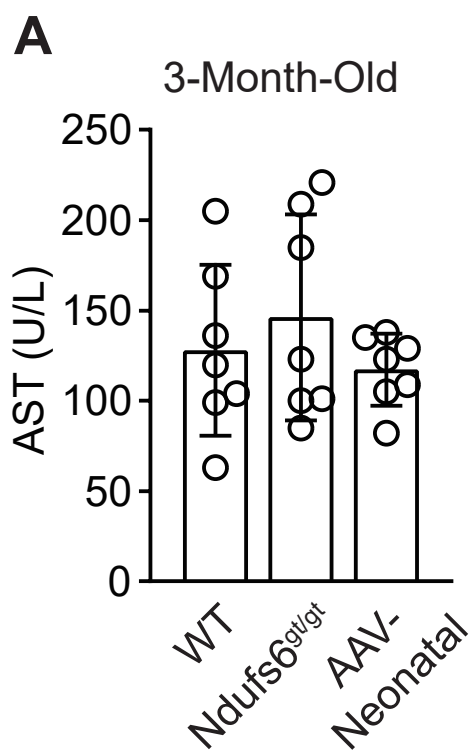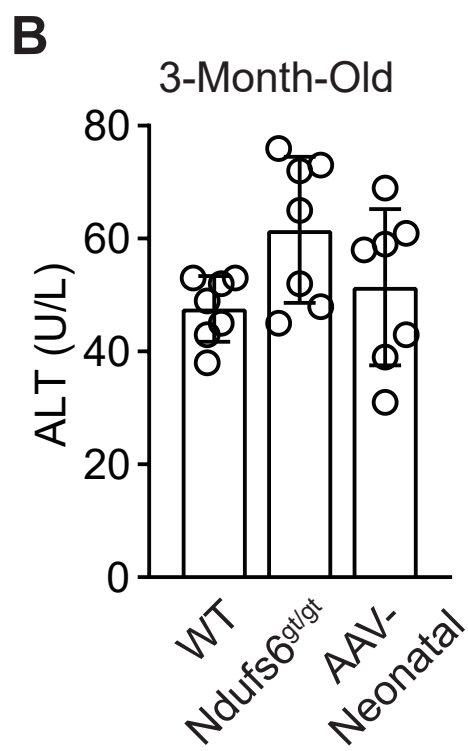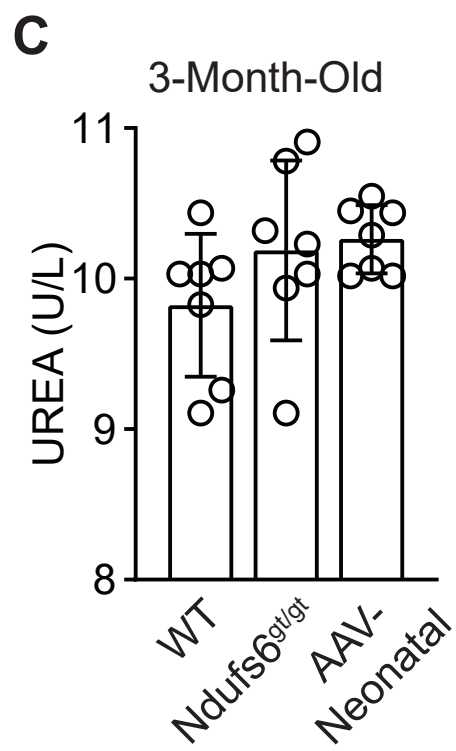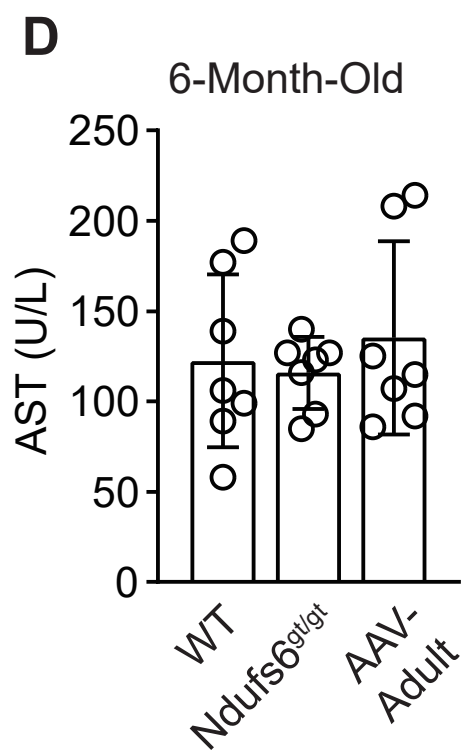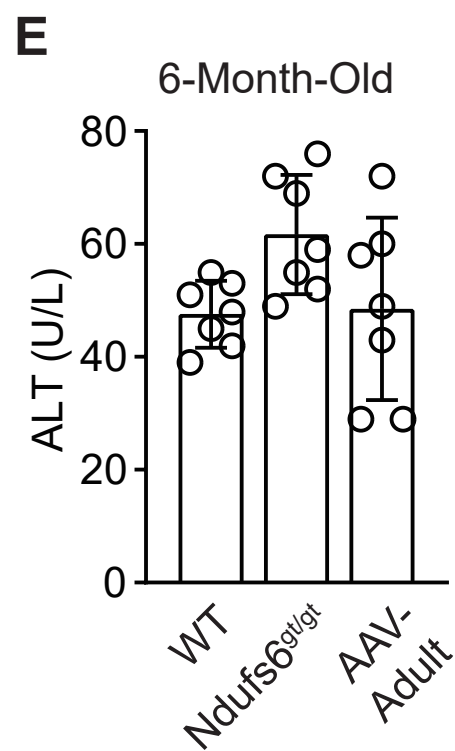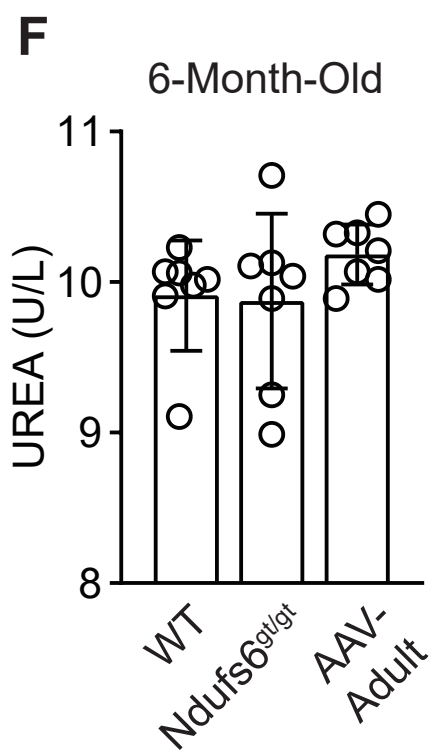

Supplement: Supplementary file 4 — Figure S4. Serum indicators of liver and kidney function after AAV gene therapy. [file 41420_2025_2524_MOESM4_ESM.pdf]

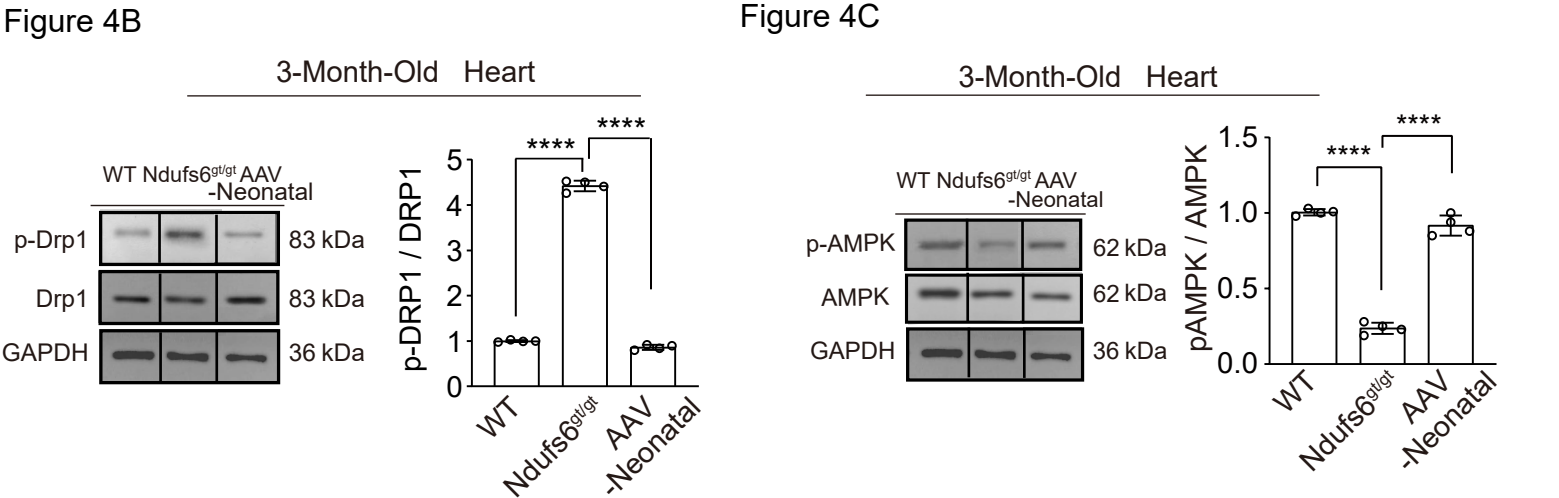

Figure 6B

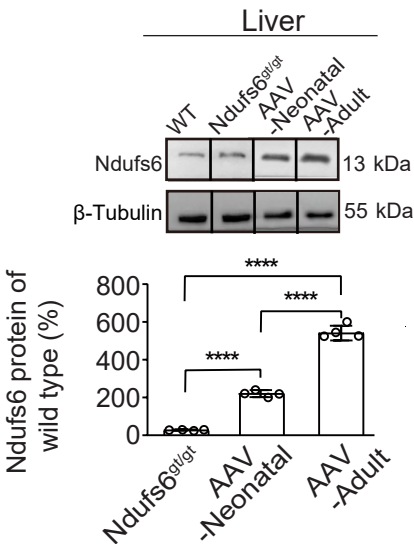

Figure 7B

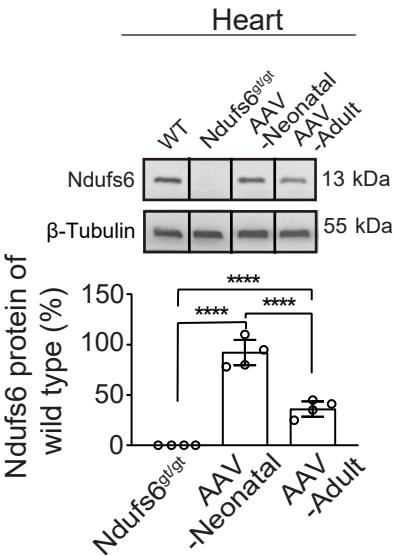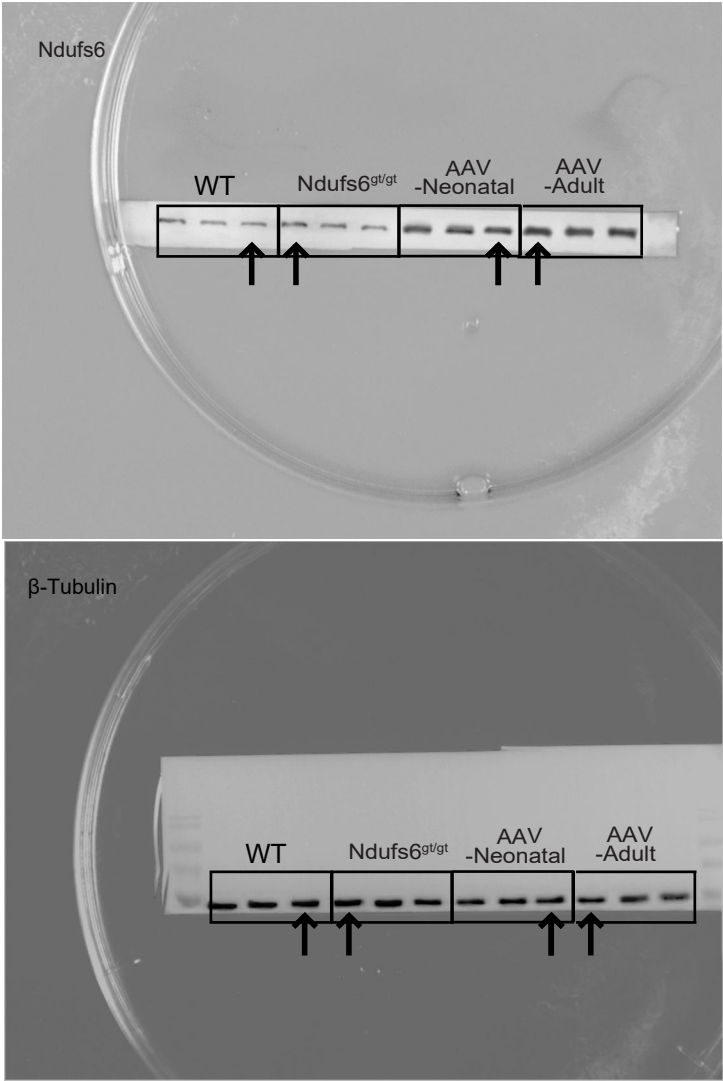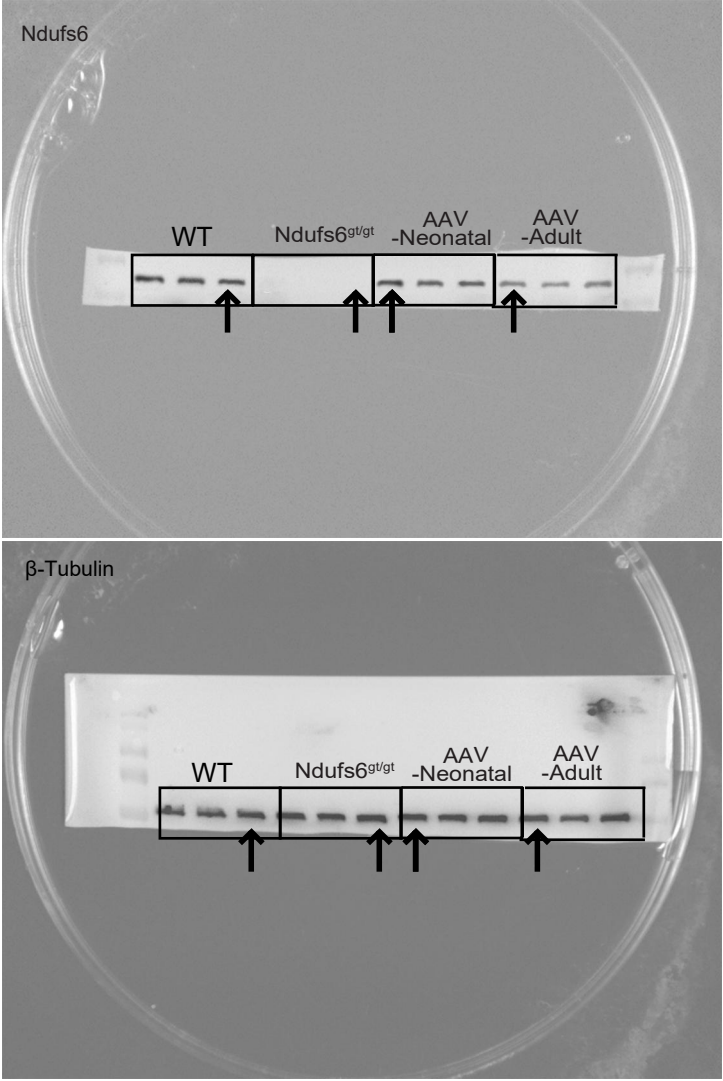

Figure 7E

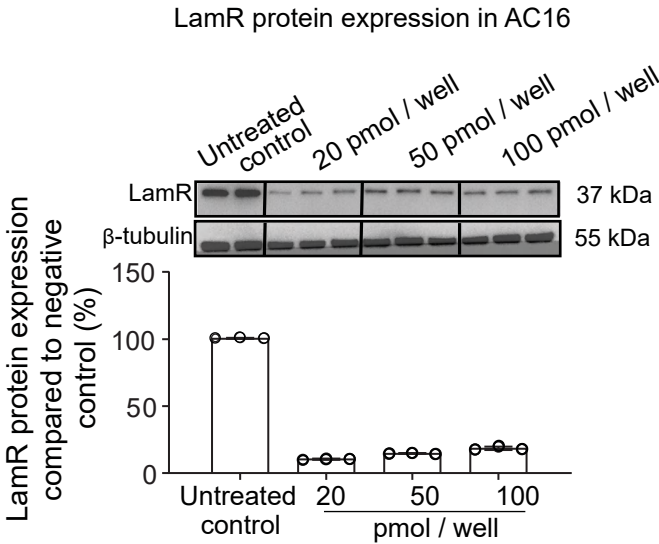

Figure 7H

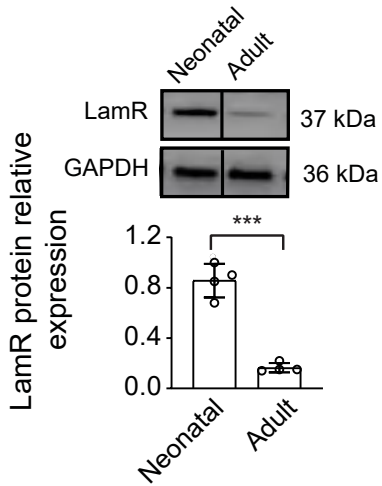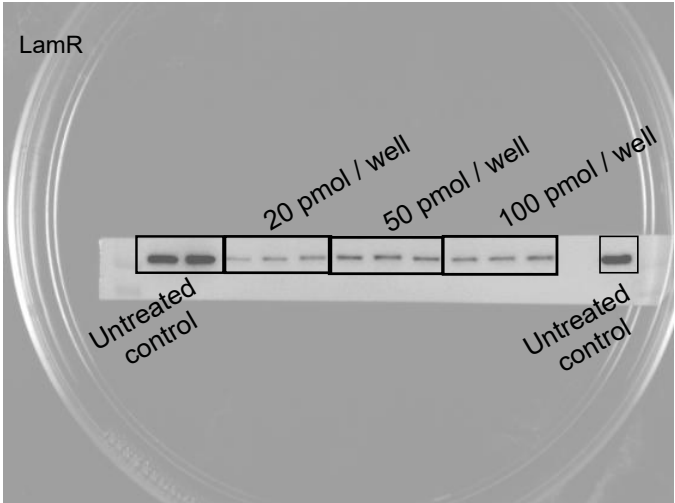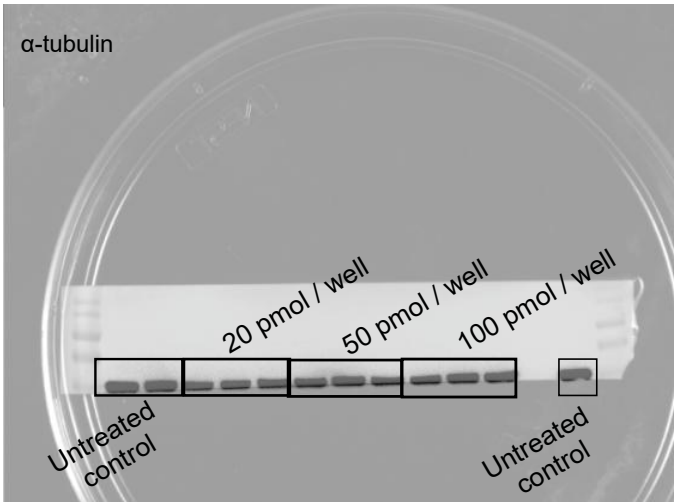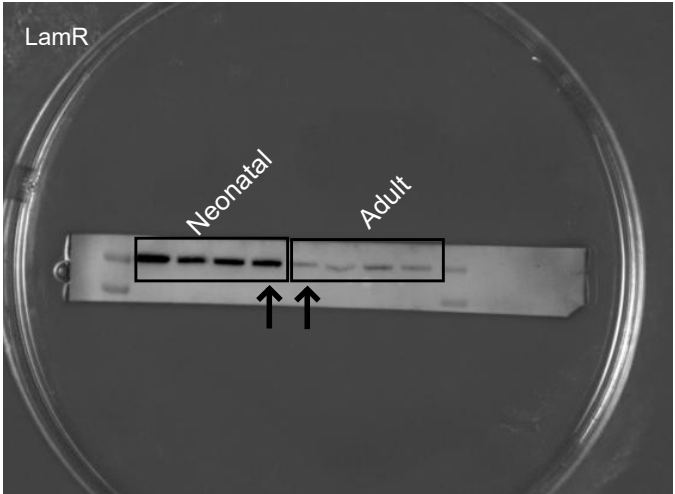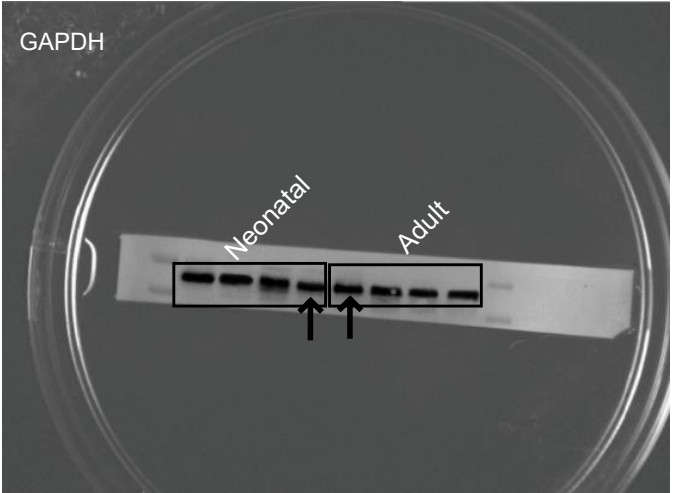

Supplement: Supplementary file 6 — Supplemental figures of western blot. [file 41420_2025_2524_MOESM6_ESM.pdf]
