# Supplementary material for: Gene therapy prevents onset of mitochondrial cardiomyopathy in neonatal mice with Ndufs6 deficiency
Source: Cell Death Discov. 2025 May 22;11:249. doi: 10.1038/s41420-025-02524-7 (PMC12095822; doi:10.1038/s41420-025-02524-7)
Supplement: Supplementary file 5 — Supplementary legends [file 41420_2025_2524_MOESM5_ESM.docx]

**Figure S1. Genotyping and *Ndufs6* mRNA expression and differential gene expression in transcriptomics of Ndufs6^gt/gt^ mice.**

(A) Body weight change from 5 to 25 weeks old in WT and Ndufs6^gt/gt^ mice (n=10). * *p* =0.0382, ** *p* =0.0038, *** *p* =0.0002. (B) Genotyping results of Ndufs6^gt/gt^ mice. (C) *Ndufs6* mRNA expression in the heart of Ndufs6^gt/gt^ mice. (n=6). (D) Volcano plot depicting the DEGs between the WT and Ndufs6^gt/gt^ groups. The x-axis represents the log_2_FC in gene expression between the two groups, with positive values indicating upregulation in the WT group and negative values indicating downregulation. The y-axis represents the -log_10_ p-value, with higher values indicating greater statistical significance. Genes that meet the criteria of |log_2_FC| ≥ 1 and p-value < 0.05 are highlighted as significant DEGs. Points in the upper right corner represent genes significantly upregulated in the WT group, while those in the upper left corner represent genes significantly downregulated (n=3). (E) Bubble plot depicting the GO enrichment analysis of downregulated genes in the Ndufs6^gt/gt^ group compared to the WT group. The x-axis represents the gene ratio, defined as the proportion of downregulated genes associated with each GO term relative to the total number of downregulated genes. The y-axis lists the enriched GO terms. The size of each bubble corresponds to the number of genes involved in that GO term, and the color intensity reflects the significance level (e.g., -log_10_ p-value). Larger, darker bubbles indicate more significant and more highly enriched GO terms, highlighting key biological processes affected in the Ndufs6^gt/gt^ group. (F) Bubble plot of the KEGG enrichment analysis revealed associated pathways associated with downregulated genes in the Ndufs6^gt/gt^ group compared to WT group. The x-axis represents the gene ratio, defined as the proportion of downregulated genes associated with each KEGG term relative to the total number of downregulated genes. The y-axis lists the enriched KEGG terms. The size of each bubble corresponds to the number of genes involved in that KEGG term, and the color intensity reflects the significance level (e.g., -log_10_ p-value). Larger, darker bubbles indicate more significant and more highly enriched KEGG terms, highlighting key biological processes affected in the Ndufs6^gt/gt^ group.

**Figure S2. Biodistribution of AAV-eGFP in WT adult mice.**

(A) AAV-eGFP was detected in liver four weeks post tail injection in WT mice. (B) Representative images of liver, heart, spleen and brain from AAV-eGFP injected mice. Scale bar = 100μm. (C) Representative images of *eGFP* mRNA expression in AAV-eGFP delivered WT mice.

**Figure S3. AAV-hNdufs6 prevent heart dilation and restore mitochondrial function in neonatal mice.**

(A) DHE staining of ROS and quantification, (n=4, **p* = 0.0478). Scale bar = 50 um. (B) Representative ultrastructure images from electron microscopy showed mitochondrial morphology in heart tissue from WT, Ndufs6^gt/gt^ and AAV-Adult mice on 6-month-old (n=4). Quantitative measurement of mitochondrial length and proportion of fragmented mitochondria. Scale bar = 1μm. (**p* = 0.0455) (C) Mitochondrial CI activity of WT, Ndufs6^gt/gt^, and AAV-Adult groups (n=6). (D) Percentage survival of Ndufs6^gt/gt^ and AAV-Adult group (n=15).

**Figure S4.** **Serum indicators of liver and kidney function after AAV gene therapy.**

No significant differences were detected in AST (A, E), ALT (B, E) or UREA (C, F) among the groups (n=7).
